# Inhalation of Silver Nanomaterials—Seeing the Risks

**DOI:** 10.3390/ijms151223936

**Published:** 2014-12-22

**Authors:** Ioannis G. Theodorou, Mary P. Ryan, Teresa D. Tetley, Alexandra E. Porter

**Affiliations:** 1 Department of Materials and London Centre for Nanotechnology, Imperial College London, Exhibition Road, London SW7 2AZ, UK; E-Mails: ioannis.theodorou10@imperial.ac.uk (I.G.T.); m.p.ryan@imperial.ac.uk (M.P.R.); 2 National Heart and Lung Institute, Imperial College London, Cale Street, London SW3 6LY, UK; E-Mail: t.tetley@imperial.ac.uk

**Keywords:** silver nanomaterials, lung, toxicity, characterization, imaging, pulmonary surfactant

## Abstract

Demand for silver engineered nanomaterials (ENMs) is increasing rapidly in optoelectronic and in health and medical applications due to their antibacterial, thermal, electrical conductive, and other properties. The continued commercial up-scaling of ENM production and application needs to be accompanied by an understanding of the occupational health, public safety and environmental implications of these materials. There have been numerous *in vitro* studies and some *in vivo* studies of ENM toxicity but their results are frequently inconclusive. Some of the variability between studies has arisen due to a lack of consistency between experimental models, since small differences between test materials can markedly alter their behaviour. In addition, the propensity for the physicochemistry of silver ENMs to alter, sometimes quite radically, depending on the environment they encounter, can profoundly alter their bioreactivity. Consequently, it is important to accurately characterise the materials before use, at the point of exposure and at the nanomaterial-tissue, or “nanobio”, interface, to be able to appreciate their environmental impact. This paper reviews current literature on the pulmonary effects of silver nanomaterials. We focus our review on describing whether, and by which mechanisms, the chemistry and structure of these materials can be linked to their bioreactivity in the respiratory system. In particular, the mechanisms by which the physicochemical properties (e.g., aggregation state, morphology and chemistry) of silver nanomaterials change in various biological milieu (*i.e.*, relevant proteins, lipids and other molecules, and biofluids, such as lung surfactant) and affect subsequent interactions with and within cells will be discussed, in the context not only of what is measured but also of what can be visualized.

## 1. Introduction

Silver nanoparticles (AgNPs) are a class of metallic particles with at least one dimension less than 100 nanometres. The well-known antibacterial properties of AgNPs [1,2,3,4], as well as the unique optoelectronic properties of this material [5,6], has led to a dramatic increase in research and industrial production of silver nanomaterials. Among over 1800 commercially available products, identified as containing nanomaterials according to manufacturers’ reports, about 25% contain silver nanomaterials [7]. The available products range from electronic and photonic devices (such as solar panels) to textiles, food storage containers, antiseptic and antibacterial sprays and more.

The increase in the number of products containing silver nanomaterials has led to growing concerns about the potential adverse effects on human health upon exposure to Ag nanomaterials. As a result, nanosilver has been the subject of intensive investigation during the last decades. Elemental silver was considered to be of low toxicity to humans, although several cases have been reported of argyria (irreversible pigmentation of the skin) or argyrosis (irreversible pigmentation of the eyes) after chronic ingestion of colloidal silver [8,9]. The toxicity of AgNPs has been demonstrated for several species of vertebrates, invertebrates, and prokaryotic and eukaryotic microorganisms, as well as mammalian cell lines [2,10]. The toxicological outcomes upon exposure to Ag nanomaterials include oxidative stress, lipid peroxidation, inhibition of mitochondrial activity, damage of DNA, and cell apoptosis [11,12,13,14,15,16,17,18,19,20]. AgNPs are manufactured in several different formats, which may lead to varied toxicological potentials. Several physicochemical properties of the AgNPs have been shown to play a role in their bioreactivity (e.g., shape [16,21], size [12] and coating agent [14]) but the exact mechanisms remain elusive. Several studies have linked the toxicity of AgNPs to their dissolution and the release of free Ag^+^ ions [20]. Silver is an oxidation catalyst and undergoes slow oxidative dissolution as Ag^+^ so the chemical nature of AgNPs in the environment changes with time (depending on the environment), therefore both size and oxidation state will critically determine any cytotoxicity. Release of silver ions typically relies on the presence of dissolved molecular oxygen [22], and, given the presence of molecular oxygen, the amount and rate of silver ions released will increase as the pH becomes more acidic [23]. The kinetics of silver ion release will depend on the size [24] and surface functionalization of the NPs [25], temperature [24,26] and composition of the surrounding media [27,28,29]. Once released, the Ag^+^ ions will react with other species in their environment and may precipitate as insoluble silver compounds or undergo complexation with proteins.

**Figure 1:**
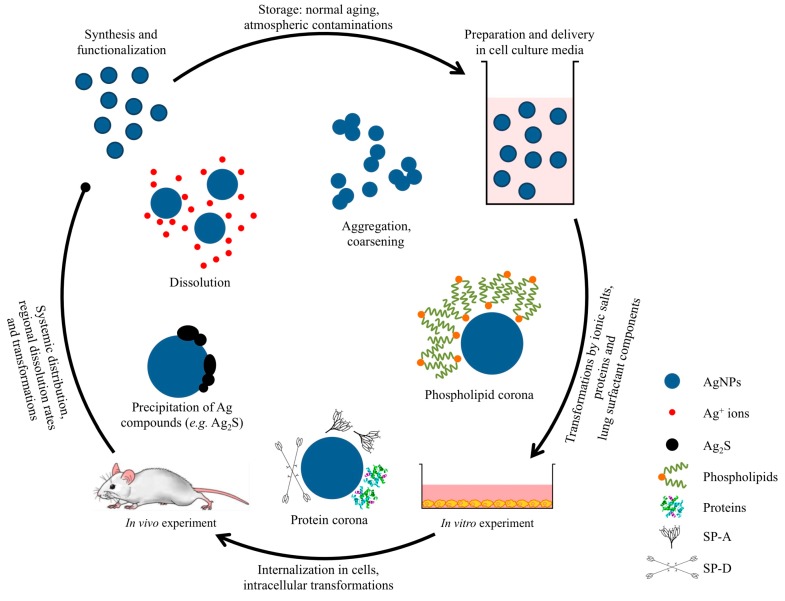
Examples of transformations to the physicochemical properties of silver nanoparticles (AgNPs). To elucidate the mechanisms of biological action of AgNPs, these transformations must be carefully considered and comprehensive characterization should take place at each stage of the *in vitro* and *in vivo* testing.

There has been much discussion in the literature as to whether the reactivity of AgNPs arises due to an ionic [27,30] or particulate effect [31,32,33,34], or both. Attempts have been made to resolve this question by incubating cells with AgNO_3_ or by culturing cells in anaerobic conditions [30]. However, several discrepancies still exist between the published results. This is most likely caused by the lack of sufficient controls over the particles used and the cellular systems investigated, making it hard to compare between experiments performed by different groups. On the one hand, further efforts are required to understand the stability of AgNPs and the kinetics of Ag^+^ ion release in biological environments. AgNPs are highly dynamic and their properties can change drastically when incubated in biological media, leading for example to aggregation or the formation of biomolecule coronas (Figure 1). As a result, characterization of as-synthesized AgNPs alone is not enough to predict their biological activity but appropriate characterization should also take place at conditions that realistically simulate *in vivo* exposure scenarios. When combined with molecular biological studies, this information can provide greater insight into the unique ENM behaviour and a better appreciation of potential effects on human health and the environment. Since experimental techniques commonly used in the past for AgNP characterization, such as atomic absorption spectroscopy or dynamic light scattering, may possess limitations in detecting transformations of the physicochemical properties of AgNPs in different environments (e.g., pulmonary surfactant), several complementary techniques need to be applied. On the other hand, it is becoming increasingly clear that the extracellular release of Ag^+^ ions by AgNPs cannot wholly account for the observed toxicity. Additional effects at the particle-cell membrane interface and inside cells seem to play a role in the biological action of AgNPs [35]. Therefore, a synergistic effect between AgNPs and Ag^+^ ions must be considered in order to obtain accurate conclusions about the mechanisms of toxicity. The focus should be placed on developing new metrology methods that will be able to link the existing discrepancies between the effects of AgNPs and Ag^+^ ions. New approaches based on the correlative application of high spatial and energy resolution analytical microscopy techniques may offer an improved understanding of the mechanisms by which AgNPs interact with cells, and can guide the selection of the most relevant toxicological assays to test. Imaging and analysis could also help to determine whether the toxicological findings relate directly to the localization of AgNPs inside cells, or whether they are more general. Finally, the development of new methods for the quantification of Ag^+^ ions released intracellularly will prove invaluable in discriminating between the effects of AgNPs and Ag^+^ ions.

## 2. Pulmonary Exposure to Silver Nanoparticles

An increase in the number and production volume of products containing AgNPs will lead to a larger release into the environment during manufacture [36], use, washing or disposal of the products. There is currently very little data on the magnitude of release of AgNPs but efforts have begun to provide quantitative estimations of the environmental concentrations of engineered nanomaterials [37,38]. During manufacturing, AgNPs can be present in either powder or liquid formats and potentially present a health risk to workers. In an industrial manufacturing facility, significant release of AgNPs was observed during processing as soon as the reactor, dryer and grinder were opened, leading to a possible occupational exposure even for wet production processes [39]. In a laboratory setting, handling of silver nanomaterial powders inside a fume hood, led to an increase in the number concentration of particles in the breathing zone of a worker [40]. Moreover, few data exist on the effects of exposure of consumers to NPs in realistic application scenarios relating to the use of nanotechnology-based consumer products. As two studies have shown, the use of sprays containing AgNPs can lead to the generation of nanosized aerosols and the release of NPs near the human breathing zone [41,42]. Moreover, Ag is imbedded in textiles in a variety of different forms, such as ionic Ag, AgCl or metallic AgNPs, to provide them with an enhanced antimicrobial activity. These silver-treated textiles can be a source of AgNPs in the washing solution when laundering fabrics, regardless of the initial form of treatment [43]. Consequently, AgNPs are a potential occupational, environmental and public hazard and their effects must be assessed before the production and use of these nanomaterials are spread more widely. Regulatory frameworks to protect both public health and the environment are still under development. Therefore, scientific evidence is needed in order to inform these decisions and allow for the establishment of risk assessment processes.

Human exposure to AgNPs may take place through various routes, including the respiratory tract, the skin, the gastrointestinal tract, the reproductive system or the circulatory system (Table 1) [1,4,44]. For instance, the use of AgNPs in wound dressings, antibacterial textiles, or cosmetic products could increase human skin exposure while AgNPs used in food packaging and kitchen utensils could lead to ingestion of AgNPs. Moreover, exposure of the reproductive system could occur through the use of contraceptive devices or hygiene products containing AgNPs. Another important uptake route is the circulatory system, following intravenous injection of AgNP-based drugs or drug delivery/diagnostic systems. Although all these scenarios need to be assessed as potential portals of entry, inhalation of AgNPs is considered as one of the most important routes of exposure, since the respiratory system serves as a major portal for ambient particulate materials. Pathologies resulting from airborne particle materials at the micron level, such as quartz, asbestos and carbon, have been topics thoroughly researched in occupational and environmental medicine and associations between ultrafine particle inhalation and increased cardiovascular and pulmonary morbidity and mortality have been made [45,46]. Recently, the pathogenic effects of inhaled manufactured nanoparticles have also received attention [44,47]. Several healthcare, hygiene and antibacterial spray products containing AgNPs have now entered daily use. Therefore, AgNP aerosols directly applied into the nasal or oral cavity (e.g., nasal drops for rhinitis treatment) are of great concern, as concentrated NPs can be channeled into the lungs. The increasing use of sprays containing AgNPs, such as deodorants, shoe sprays or disinfectant cleaning products could also lead to accidental AgNP inhalation. Finally, as described above, AgNPs pose a risk of occupational respiratory exposure in manufacturing and research facilities.

**Table 1:** Possible routes of human exposure to silver nanoparticles.

Route of Exposure	Sources of Exposure
Respiratory System	Handling AgNPs in manufacturing or research facilities;
	Aerosols directly applied in the nasal or oral cavities;
	Sprays (e.g., deodorants, shoe sprays, cleaning products);
	Air filters, breathing masks;
	Ambient airborne AgNPs
Skin	Wound dressings;
	Antibacterial textiles (e.g., sheet, towels, socks, underwear, fitness wear);
	Antibacterial surfaces, paints;
	Cosmetic products (e.g., lotions, roll-on deodorants, hair products);
	Computer hardware and mobile devices
Gastrointestinal Tract	Food packaging, cooking utensils and coatings;
	Water filters;
	Health supplements;
	Oral hygiene products (e.g., toothpastes, toothbrushes)
Reproductive System	Contraceptive devices;
	Feminine hygiene products
Circulatory System	Intravenous injection of AgNP-enabled drugs or drug delivery/diagnostic systems;
	Implants, medical catheters

The human lungs consist of approximately 2300 km of airways and 300 million alveoli, giving rise to a surface area of about 150 m^2^. This vast internal surface area facilitates a broad access of inhaled materials to the lung, which cannot always deal adequately with the wide range of airborne materials present in urban or occupational environments [45]. The most critical characteristic determining the deposition patterns of particulates in the respiratory tract is the particle size distribution [45]. Larger particles (5–30 μm) are usually deposited in the nasopharyngeal region by the inertial impaction mechanism and smaller particles (1–5 μm) in the tracheobronchial region, mainly due to sedimentation. These particles are typically cleared by bodily responses, such as coughing, mucociliary transport and/or phagocytosis by alveolar macrophages. The major deposition mechanism for particles smaller than 0.5 μm is diffusion, therefore NPs can penetrate deeply into the alveolar region, where clearance may be insufficient [45,48,49]. The deeper the particle deposition, the longer it takes for pulmonary particle clearance; it is believed that this might give rise to a higher probability of adverse health effects due to continuous particle-tissue interactions [50]. Additionally, phagocytosis by alveolar macrophages to remove inhaled NPs appears to be less efficient than for larger particles [51,52]. Therefore, NPs can effectively access the alveolar region of the lungs and come into contact with the alveolar epithelium, where they can possibly be taken up by epithelial cells and fibroblasts. Once AgNPs reach the alveoli, further barriers to diffusion into the blood circulation are limited. This is because the epithelium that separates the inhaled air from the blood capillaries is very thin (<0.5 μm), consisting of a monolayer of type I and type II epithelial cells [53]. Type I (squamous alveolar) cells form the structure of the alveolar wall, whereas type II (cuboidal alveolar) cells continuously release pulmonary surfactant by exocytosis. Any cellular or protein damage in this region could not only have an impact on pulmonary homeostasis but would also determine possible translocations of AgNPs to other organs and allow them to elicit toxic effects at extrapulmonary sites. Hence, the interactions of AgNPs with the human lung epithelium urgently needs to be addressed, in order to predict their adverse effects, provide guidelines for their safe use and direct the regulation of silver nanomaterials.

## 3. Bioreactivity of Silver in the Lung

### 3.1. The Bioreactivity of AgNPs in the Lung in Vivo

Although AgNPs have been identified as a possible exposure hazard, there is very limited information about how this class of nanomaterial interacts with the lung *in vivo*. Nanotoxicology assessment studies performed *in vivo* using the appropriate dosing amounts and routes of exposure can provide useful information because of the diversity of systemic phenotypic response and the anatomic influence, which can be directly translatable from animal models to human exposures. Particle pharmacokinetics combined with studies of regional dissolution rates can provide insights into the impact of dosing on systemic physiology and positional anatomy [54]. For instance, inhalation and instillation of 15 nm AgNPs in rats showed that lung particle content rapidly decreased following inhalation. The AgNPs were subsequently detected in the blood and other organs, such as the liver, kidney and brain [55]. In several cases, a gender-dependent difference for AgNP accumulation in kidneys has been reported, with females exhibiting a higher concentration than males [56,57,58]. Another study on the distribution and accumulation of AgNPs in rats following subcutaneous injection also revealed that the particles had translocated to the blood circulation and distributed throughout the main organs, especially in the kidney, liver, spleen, brain and lung [59]. Ultrastructurally, AgNPs that had accumulated in organs were observed within different types of cells, such as renal tubular epithelial cells and hepatic cells. Moreover, AgNPs induced blood-brain barrier destruction and astrocyte swelling, and caused neuronal degeneration [59]. In most studies that report on the anatomic distribution of silver following inhalation of AgNPs *in vivo*, silver is quantified by inductively coupled plasma mass spectrometry (ICP-MS) due to its high sensitivity [55,57,59]. However, this technique requires the digestion of tissue samples by strong acids and therefore ICP-MS detects the total elemental silver content of samples. Consequently, it is difficult to draw conclusions about the form of silver that has reached each organ, *i.e.*, distinguish between metallic AgNPs, ionic Ag^+^ or Ag compounds. Other groups have employed the silver-enhancement method to track Ag in tissues [58,60], which is based on the autometallography (AMG) technique [61]. This method is based on the principle that small-sized clusters of silver enhance the reduction of silver ions that are added on their surface, resulting in an increase in the size of the clusters. These can become large enough to be viewed under a light microscope, thus confirming the presence of Ag in tissue, but without being able to characterize the morphology or chemistry of the original AgNPs. Moreover, this method presents several drawbacks that could lead to artefacts. For example, autonucleation of the enhancement solution may lead to the formation of nanoparticles that are subsequently catalytic for further growth.

A 28-day inhalation toxicity study of AgNPs on rats by Ji *et al*., showed no significant changes in the haematology and blood biochemistry in either the male or female rats [62]. To further evaluate these findings, a sub-chronic 90-day inhalation study was performed by the same group [56]. Among the lung function test measurements, the tidal volume and minute volume showed a statistically significant decrease during the 90 days of AgNP exposure. Moreover, histopathological examinations indicated dose-dependent increases in lesions related to AgNP exposure, such as mixed cell infiltrate and chronic alveolar inflammation, including thickened alveolar walls and small granulomatous lesions. Their findings indicated that prolonged exposure to AgNPs can induce lung function changes, along with inflammation, at much lower mass dose concentrations, when compared to Ag microparticles. In contrast, in a 28-day inhalation exposure performed by Hyun *et al.*, histopathological examination showed that the nasal cavity and lungs from the AgNP-exposed groups exhibited no “remarkable” changes compared to the control group [63].

In contrast, Lee *et al.*, found that a 14-day inhalation exposure of mice to AgNPs led to alterations in brain gene expression [64]. A total of 468 genes in the cerebrum and 952 genes in the cerebellum were identified as AgNP-responsive. The largest groups of gene products affected by AgNP exposure included 73 genes in the cerebrum and 144 genes in the cerebellum. AgNP exposure modulated the expression of several genes associated with motor neuron disorders, neurodegenerative disease, and immune cell function, indicating potential neurotoxicity and immunotoxicity associated with AgNP exposure [64]. In a recent 90-day inhalation study it was demonstrated that a decrease in lung function and pulmonary inflammation could persist even after termination of the exposure in male rats, while in females there was no effect on lung function and a gradual improvement in lung inflammation following cessation of exposure [65].

While the results of the above studies clearly demonstrate a pro-inflammatory potential of AgNPs in the lung, the mechanism through which lung injury occurs and the characteristics of AgNPs that contribute to lung injury have not been assessed systematically. Recently, Wang *et al.*, compared the effects of 20 and 110 nm AgNPs with two different surface coatings, citrate and polyvinylpyrrolidone (PVP), on mice [60]. Both size and surface coating were found to affect the cellular toxicity of AgNPs as well as their acute *versus* sub-chronic lung injury potential. Smaller particles induced more cellular toxicity and oxidative stress than the larger particles, possibly due to a higher rate of dissolution and Ag^+^ ion bioavailability. Moreover, there was a higher propensity for citrate-20 nm AgNPs (C20) to generate acute neutrophilic inflammation in the lung and to produce chemokines compared to citrate-110 nm AgNPs (C110). In contrast to the more intense acute pulmonary effects of C20, C110 induced mild pulmonary fibrosis at day 21, likely as a result of slow but persistent Ag^+^ release, leading to a sub-chronic injury response [60]. Further investigation is therefore necessary in order to elucidate the mechanisms through which the physicochemical properties of inhaled AgNPs affect their interactions with the lung. Additionally, understanding how the impact of AgNPs at the cellular level *in vitro* translates to their *in vivo* toxicological potential will be essential for the development of a predictive toxicological paradigm for AgNP toxicity, the design of accurate screening assays and ultimately the development of safer products.

Although the importance of dosimetry in biological studies is well-known, the relevance of AgNP doses used in animal inhalation studies to realistic exposure scenarios has rarely been assessed. This is in part due to the fact that very few data on AgNPs are currently available to evaluate occupational and consumer exposure. The recommended threshold limit value (TLV) set by the American Conference of Industrial Hygienists (ACGIH) for AgNP inhalation is 100 μg/m^3^ [66,67], while the recommended exposure level (REL) for nano-Ag by the US National Institute for Occupational Safety and Health (NIOSH) is 10 μg/m^3^ [68]. A study on airborne NP exposures associated with the manual handling of nanosilver in fume hoods showed a peak airborne concentration of 7000 particles/cm^3^ [69]. Another assessment reported a similar particle number concentration in the workplace air, while mass concentrations were between 0.02 and 1.02 μg/m^3^ [70]. A recent study estimated the potential exposure of workers in nanosilver manufacturing facilities by performing personal sampling, area monitoring and real-time monitoring over three days [71]. The highest AgNP concentrations were obtained from area sampling in the injection room, ranging from 5.01 to 288.73 μg/m^3^. These values were used in the study by Wang *et al*., to calculate the relevant doses to administer in their animal experiments [60]. Assuming a monthly deposition in human lungs, the equivalent dose per mouse was estimated at 0.33 mg/kg, therefore 0.1, 0.5 and 1.0 mg/kg were selected as the dose range to perform bolus instillation studies. Based on their findings, they predicted that a human lung burden equivalent to a bolus dose of 0.1–1.0 mg/kg in a mouse could be associated with incremental acute pulmonary inflammation, whereas a lung burden equivalent to 1.0 mg/kg in a mouse may lead to sub-chronic pulmonary effects in a human [60]. For comparison, the doses used in other *in vivo* studies are summarized in Table 2.

**Table 2:** Exposure doses used in *in vivo* studies of silver nanoparticles.

Species	Size (nm)	Dose	Exposure Method	Exposure Time	Reference
C57BL/6 Mice, 8 weeks old	20, 110	0.1, 0.5 and 1.0 mg/kg	Oropharyngeal aspiration	Single injection	[60]
Sprague-Dawley rats, 6 weeks old	15	0.66 × 10^6^ particles/cm^3^ (49 μg/m^3^) 1.41 × 10^6^ particles/cm^3^ (117 μg/m^3^) 3.24 × 10^6^ particles/cm^3^ (381 μg/m^3^)	Inhalation	6 h/day, 5 days/week, for 12 weeks	[65]
Sprague-Dawley rats, 8 weeks old	18	0.7 × 10^6^, 1.4 × 10^6^ and 2.9 × 10^6^ particles/cm^3^	Inhalation	6 h/day, 90 days	[72]
Wistar rats, 8–10 weeks old	15–40	4, 10, 20 and 40 mg/kg	Intravenous injection	32 days (injected at 5 day intervals)	[73]
C57BL/6 mice	20	1.91 × 10^7^ particles/cm^3^	Inhalation	6 h/day, 5 days/week, for 2 weeks	[64]
Wistar rats, female	50–100	62.8 mg/kg	Subcutaneous injection	Single injection	[59]
C57BL/6N mice, adult male	30	100, 500 and 1000 mg/kg	Intraperitoneal injection	24 h	[74]
Sprague-Dawley rats, 6 weeks old	55	0.7 × 10^6^, 1.4 × 10^6^ and 2.9 × 10^6^ particles/cm^3^	Inhalation	6 h/day for 90 days	[56]
Sprague-Dawley rats, 8 weeks old	13–15	1.73 × 10^4^ particles/cm^3^ (0.5 μg/m^3^) 1.27 × 10^5^ particles/cm^3^ (3.5 μg/m^3^) 1.32 × 10^6^ particles/cm^3^ (61 μg/m^3^)	Inhalation	6 h/day, 5 days/week, for 4 weeks	[63]
Sprague-Dawley rats, 8 weeks old	15	1.73 × 10^4^,1.27 × 10^5^ and 1.32 × 10^6^ particles/cm^3^	Inhalation	6 h/day, 5 days/week, for 4 weeks	[62]
Fischer 344 rats, female	15	3 × 10^6^ particles/cm^3^ (133 μg/m^3^)	Inhalation	6 h	[55]

### 3.2. The Bioreactivity of AgNPs in Vitro

*In vitro* studies offer a relatively straightforward method to determine which features of a nanosubstance could predict an increased cellular reactivity. *In vitro* testing is one of the major options suggested in recent proposals on strategic approaches to discover any undesirable effects of NPs or to study NP cell targeting. However, the lack of standardized procedures for the evaluation of NP toxicity makes it difficult to compare between studies performed by different laboratories. One of the factors that can lead to inconsistencies between different studies is the use of different cell types, because the toxic effects of NPs are highly dependent on the type of cell encountered [75]. This is due to the variation in cell physiology (e.g., epithelial or macrophage cells), proliferation state (tumoral or resting cells), membrane characteristics and phagocyte characteristics among different cell types [76]. For example, cancer cell lines can be more resilient towards NP toxicity than normal cells due to an increased rate of proliferation and metabolic activity [77,78]. Also, since membrane transport depends on the composition of cellular membranes, cell type can greatly influence the amount of uptake of NPs into cells as well as their fate in the intracellular environment. Therefore, in order to identify the exact effect of NPs on the organs or cells of interest, the *in vitro* study should include cells that represent the relevant exposure scenario. Since NPs will encounter different cell types depending on the exposure route, different levels of toxicity can be expected with different exposure routes. Consequently, numerous cell types ranging from endothelium, blood, spleen, liver, nervous system, heart and kidney are all of interest in toxicity studies of NPs in general and AgNPs in particular.

In summary, a variety of oxidative stress-related changes have been observed in various mammalian cell types after exposure to AgNPs [79,80]. For example, the generation of reactive oxygen species (ROS), is induced after the treatment of cells with AgNPs at concentrations as low as 0.2 μg/mL in human glioblastoma cells (U251) [20], human Jurkat T cells [81], human colon cancer cells (HCT116 and HT29) [82,83], human hepatoma cells (HepG2) [84,85], human Chang liver cells [19], mouse germline stem cells (C18-4) [86,87], mouse fibroblasts (NIH3T3) [82], mouse neuroblastoma cells (N2A) [88], rat liver-derived cell line (BRL 3A) [89] and rat pheochromocytoma cells (PC12) [90]. The level of lipid peroxidation is also increased by AgNP treatment in human skin carcinoma and fibrosarcoma cells [17], as well as in Chang liver cells [19]. Moreover, biochemical and molecular changes related to genotoxicity have been found following AgNP exposure in a variety of cells [19,20,81,84], including human mesenchymal stem cells [91], human hepatocyte cell line (L02) [92] and mouse embryonic stem cells and embryonic fibroblasts [93]. Furthermore, AgNP-induced apoptosis has been demonstrated in human-derived cells such as colon cancer [83,94] and hepatoma cells [85] as well as in Jurkat T [81] and HeLa cells [95]. Evidence of apoptosis has also been provided in some animal-derived cells, including mouse blastocysts [96], NIH3T3 and L929 fibroblasts [82,97], embryonic fibroblasts and stem cells [93] and baby hamster kidney cells [94]. As far as the lungs are concerned, various cells have been used to model the epithelial barrier. Primary cells directly isolated from tissue are not preferable due to their limited life span and variations in quality (donor variations and quality of preparation) [98]. Immortalized cell lines, although not as well differentiated as primary cells, provide high reproducibility and are most often used for the assessment of cellular toxicity and permeation, with human lung adenocarcinoma-derived A549 cells being the most popular [98]. Although very useful for toxicity testing, A549 cells are less suitable to assess permeation as they do not form tight intercellular junctions. To assess the bronchial barrier, Calu-3, 16HBE14o-, and BEAS-2B cells are usually used. The HPV-E6/E7 and hTERT immortalized bronchial epithelial cell line NuLi-1 has recently been employed as a model for the bronchial epithelial barrier [99]. Finally, models for the alveolar barrier use either primary or immortalized AT-II cells or NCI-H441 cells. Despite the fact that numerous studies have been published, investigating a wide of range cells that represent different exposure routes, including inhalation, the determination of a trend for AgNP toxicity can still be considered complex. In many of these studies, independent experimental methods have been employed, especially in regards to the physicochemical properties of the AgNPs tested. Consequently, it is not completely feasible to compare data from different studies, especially quantitative data such as half-maximal inhibitory concentration (IC_50_) values.

Moreover, the selection of appropriate cytotoxicity assays is vital to the accurate assessment of NP toxicity, especially since some may interfere with the actual toxic effect produced by the NP [100,101,102,103]. For instance, single walled carbon nanotubes (SWCNTs) have been shown to interact with MTT-formazan crystals formed after the reduction of MTT [100]. This led to a false cytotoxic effect of SWCNTs on A549 human alveolar epithelial cells within the MTT assay. Cytotoxic assays can provide a large amount of information about toxic endpoints, such as membrane integrity lactate dehydrogenase (LDH), cellular metabolic activity (MTT), oxidative stress (reactive oxygen species (ROS) and apoptosis (fluorescent Annexin V or caspase substrates). These assays are generally employed to measure acute toxic effects of cultured cells and, although they can be used to investigate cell viability, the results from one assay are difficult to directly compare with another as they measure different parameters [104]. AgNPs have been shown to cause various effects and interact with biological components in numerous ways, making it nearly impossible to cover the whole scale of cell-particle interactions in a single study. Correlating the information provided by toxicity assays with imaging techniques could provide very valuable information on local alterations in cell morphology, the localization of AgNPs and their transformation within cells and tissues. This would improve our understanding of the cytotoxicity mechanisms induced by AgNPs, depending on their concentration, size, shape, surface modification but also on the target cell type.

Several studies have reported morphological changes in the cell membrane and cell shape after exposure to AgNPs. Using phase contrast microscopy, Lee *et al.*, examined live cells from the human alveolar cell line A549 exposed to 10, 50 and 200 µg/mL AgNPs for 24 h. Significant morphological changes characteristic of cell death, including cell shrinkage, few cellular extensions, restricted spreading pattern, and increased floating of cells from the substrate were observed in a dose-dependent manner [105]. A rat alveolar macrophage cell line incubated with hydrocarbon-coated AgNPs of different sizes (15, 30 and 55 nm) presented size-dependent changes in cellular morphology [106]. The most pronounced changes were observed for cells treated with 15 nm AgNPs, with cell shrinkage and cellular debris floating in the medium. Cells exposed to 30 nm AgNPs presented abnormal sizes, with agglomerated AgNPs inside and outside the cells, whereas 55 nm AgNPs-treated cells had minor morphological changes [54]. Nguyen *et al.*, compared the toxicity of PVP-coated AgNPs and uncoated AgNPs in a mouse macrophage cell line (J774A.1). Both types of AgNPs induced cell damage, including shrinkage, deformation and enlargement of mitochondria. However, uncoated AgNPs resulted in cell shrinkage, whereas coated AgNPs induced cell elongation and enlargement. These different responses to AgNPs suggest different mechanisms of cell damage depending on their surface coating [107]. A systematic investigation on the cytotoxic effects, cellular response and membrane damage caused by four types of AgNPs with different surface charge was carried out on mouse macrophage (RAW-264.7) and lung epithelial (C-10) cell lines. Cytotoxicity was found to be strongly dependent on the surface charge of the AgNPs, with the positively charged AgNPs being the most toxic. The same study concluded that the response to AgNPs is dependent on the cell type, as lung epithelial cells were found to be more resistant to the AgNPs than macrophages, regardless of the surface coating [14]. This was attributed to the fact that the aggregate size of AgNPs in the cell culture medium was more than 100 nm, allowing them to be taken up more efficiently by macrophages than epithelial cells [108].

Moreover, the exposure of cells to AgNPs has been proven to induce stress responses, such as production of reactive nitrogen species (RNS) and reactive oxygen species (ROS). AgNPs have been shown to act on various cellular targets resulting in the induction of apoptosis, the stimulation of inflammatory signalling pathways or the production of free radicals, culminating in cell death [20,109]. For instance, enlargement of the mitochondria has been observed in human colonic epithelial (HT-29), mouse macrophage (J774A.1) and human macrophage (U937) cell lines, in a dose-dependent manner, after exposure to citrate or PVP coated AgNPs [107,110]. According to Asharani *et al.*, AgNPs reduced the ATP production of human lung fibroblast cells (IMR-90), causing damage resulting in altered mitochondrial respiratory chain activity, dissipation of mitochondrial membrane potential and stimulation of apoptotic pathways [20]. Structural damage of the mitochondria may in turn lead to a disruption of cellular ROS balance. Evidence in the literature suggests that AgNPs can bind to thiol containing proteins or peptides, such as GSH, restricting their availability for ROS neutralization and resulting in an oxidant-mediated response to AgNPs [31,111]. Lim *et al.*, showed that exposure of a human macrophage cell line (U937) to 5 nm PVP-coated AgNPs resulted in swelling of the mitochondria and formation of double-layered membrane structures. The authors suggested that mitochondrial swelling was due to necrotic changes in the cell and that the presence of double-layered membrane structures was related to autophagosome formation [110]. Therefore, mechanisms other than apoptosis may also be involved in the initiation of cell death. Similarly, Gliga *et al.*, reported that 10 nm polymer-grafted AgNPs led to morphological changes suggestive of autophagy in a normal human bronchial epithelial cell line (BEAS-2B) [112]. The induction of autophagy, *i.e.*, the degradation of unnecessary or dysfunctional cellular components through the action of lysosomes, has been reported for several types of NPs, including AgNPs and AgNWs, and may represent a common cellular response to NPs [113,114].

In some studies, evidence of genotoxicity upon exposure to AgNPs has also been obtained. Nanoparticles may interact with DNA chains through nuclear penetration and consequently induce DNA damage [115,116]. Occasionally, AgNPs have been found inside nuclei [15,117]. Cronholm *et al.*, [118] detected agglomerates of AgNPs (from 0.5 to 1 μm) inside the nuclei of A549 type II alveolar epithelial cell line. Due to the large size of the aggregates, compared to the size of nuclear pores, it is possible that AgNPs enter via vesicles lodged to the nucleus [20], individual particles are internalized via the nuclear pores and agglomerate inside the nucleus or AgNPs enter during mitosis [119,120]. Furthermore, a mouse macrophage cell line (RAW264.7) exposed to chitosan-coated AgNPs showed dose-dependent nuclear shrinkage and chromosome abnormalities, such as double-strand breaks [121].

The results of *in vitro* studies on the toxic responses to AgNPs have been mixed. There is an on-going debate about the properties of AgNPs that determine their interactions with cells and the extent to which their bioreactivity depends on the release of free Ag^+^ ions. Exposure of mouse macrophages (J774A.1) to citrate and PVP-coated AgNPs resulted to an increase in ROS generation after 24 h for both types of particles, suggesting that surface modification did not have a great effect on cytotoxicity [107]. Similarly, Gliga *et al.*, compared uncoated and citrate- and PVP-coated AgNPs of different primary sizes (10 up to 75 nm). They showed that only 10 nm AgNPs, regardless of surface coating, were cytotoxic on human bronchial epithelial cells (BEAS-2B). Their findings suggested that particle size was the key property determining their cytotoxicity [112]. A size-dependent toxicity of AgNPs on a rat alveolar macrophage cell line was also demonstrated by Carlson *et al.*, who found the predominant mechanism of toxicity to be mediated through oxidative stress [106]. More recently, however, Wang *et al.*, argued that both size and surface coating can affect the cellular toxicity of AgNPs, as well as their acute *versus* sub-chronic lung injury potential [60]. In their work, human bronchial epithelial (BEAS-2B) and mouse macrophage cell (RAW 264.7) lines were exposed to 20 and 100 nm PVP- and citrate-coated Ag-AuNPs (AgNPs with a gold core). The 20 nm Ag-AuNPs induced significant toxicity, in the dose range from 6.25–50 μg/mL for 24 h. Significant ROS generation, intracellular calcium flux and decline of the mitochondrial membrane potential were also measured in both cell types [60]. The higher toxicity of the smaller particles was attributed to their higher rate of dissolution and Ag^+^ bioavailability. In comparison, Herzog *et al.*, applied nebulized AgNPs to a triple cell co-culture system composed of A549 epithelial cells combined with monocyte-derived macrophages (MDM) and dendritic cells (MDDC) cultured at an air-liquid interface. In this work, however, AgNPs did not significantly increase the LDH after a 24 h exposure and the secretion of pro-inflammatory markers did not alter [122]. In our own work, Ag nanowires (AgNWs) were applied to a human alveolar type 1-like epithelial cell line for 24 h, at a dose of 25 µg/mL. Cell viability studies showed no evidence of cytotoxicity or release of reactive oxygen species [123]. Analytical transmission electron microscopy revealed the precipitation of Ag_2_S within the cell, which acts as a “sink” for free Ag^+^, significantly limiting short-term toxicological effects. The differences in toxic outcomes between these studies reflect the different chemistries and formats of the particles applied to the cells, the cell culture model and differences in cell types. An improved understanding of how the AgNPs transform within each component of the cell culture model, and whether this is representative of the lung *in vivo*, may provide improved insights into the putative pulmonary bioreactivity of AgNPs.

## 4. Evaluation of the *in Vitro* Testing of AgNPs

One of the challenges of evaluating the toxicity of engineered nanomaterials in the coming years is the development of viable alternatives to *in vivo* testing. Although several *in vitro* systems exist, providing some mechanistic understanding of AgNP toxicity, these systems will have to be optimized in the future to simulate better the *in vivo* situation and provide a holistic understanding of the “bionano” systems. Valid *in vitro* systems will lead to decreased costs compared to expensive *in vivo* studies, faster results and improved animal welfare [124]. One of the limitations of most *in vitro* studies conducted in the past is the use of single cell types, which is not physiologically relevant. Not only is the pulmonary system diverse in its cellular makeup, but also different cell types often participate in coordinated responses [125]. Rothen-Rutishauser’s group has established and evaluated an *in vitro* model of the human epithelial airway barrier composed of epithelial cells and two of the most important immune cells of the lung (macrophages and dendritic cells; derived from blood monocytes), to study NP lung-cell interactions and their possible responses [126]. Their model can be used at the air-liquid interface, allowing the direct exposure of cells to an aerosol [127], therefore representing a realistic situation following NP inhalation. Using this model, they have demonstrated that cultured epithelial cells, macrophages, and dendritic cells can cooperate in NP trafficking. Nanoparticle uptake into the cells was also enhanced in co-culture compared with monocultures [128,129]. Additionally, inhaled NPs reaching the alveoli will first come into contact with pulmonary surfactant, therefore any interactions between particles and the surfactant could have an impact on their subsequent cellular effects. However, pulmonary surfactant components are not commonly integrated in *in vitro* testing of nanomaterials. In the future, surfactant interactions should be integrated into the experimental design, to better represent the *in vivo* situation.

Moreover, in order to understand the discrepancies between the results of previous studies and elucidate the mechanisms of biological action of AgNPs, any transformations of the particles must be carefully considered at each stage of the *in vitro* experiment. More specifically, there are three steps to consider: (i) Preparation of the AgNPs and delivery to the biological medium; (ii) Transformations in the cell culture medium, either due to ionic salts or proteins, as well as the lung surfactant, prior to interaction with the cells; and (iii) Transformations of the AgNPs following internalization into the cell. This will require meticulous particle characterisation and sophisticated imaging techniques under difficult experimental conditions.

### 4.1. Preparation and Delivery to the Biological Media

The last decade has witnessed the successful synthesis of silver nanocrystals in a variety of shapes (e.g., sphere, cube, plate, rod [130]), using a wide range of experimental setups. These Ag nanocrystals can be described by a set of physical parameters that may include their size, shape, composition, structure, coating and surface charge. In principle, the properties of the nanocrystal can be tailored and fine-tuned by controlling any one of these parameters [130]. Therefore, adequate physicochemical characterization of AgNPs prior to undertaking experiments for *in vitro* toxicity assessments is paramount in order to correlate biological responses with NP properties [131]. Some published nanotoxicity studies report characteristics of the particles using only manufacturer’s data, which provides incomplete information about the particle being used. There is a wide range of methods that can be used for NP characterization. Due to its simplicity and rapidity of analysis, dynamic light scattering (DLS) is increasingly being used in many fields of science and industry for the characterization of NPs, including AgNPs [132]. However, this technique could be problematic when measuring samples with wide size distributions, multimodal distributions or containing NP aggregates. This is because, in the presence of even a low percentage of larger particles, these will dominate the light scattering signal and mask the presence of smaller particles [133,134]. Moreover, DLS may be incompatible with biological media because of the presence of various light scattering components [75]. Therefore, precise characterization of AgNPs requires the use of a combination of techniques. The key strength of electron microscopy (EM) techniques, combined with elemental analysis, is their ability to provide spatially resolved information about the morphology, size and chemistry of NPs at the same time. Sample preparation for EM may lead to drying-induced artefacts, such as NP aggregation or sample fractionation, but these limitations may be overcome by the use of cryogenic temperature EM (cryo-EM). Cryo-fixation can be applied to rapidly freeze samples, allowing cryo-EM to acquire high-resolution images of NPs, as well as cells, in their native aqueous state [135].

Even though silver is considered as a noble metal, it is far from being chemically inert. Therefore, transformation of AgNPs caused by poor experimental control will likely interfere with the prediction of silver toxicity results. Moreover, information of AgNP behaviour during their full life cycle is limited. Normal aging of the NPs (e.g., Ostwald ripening, corrosion, aggregation, surface-state modification) may already affect their properties. The atmospheric corrosion of silver is an example of transformation that could take place over poorly controlled storage conditions. Due to existing gaseous hydrogen sulphide (H_2_S), carbonyl sulphide (OCS) and carbon disulphide (CS_2_) in the atmosphere, silver sulphidises upon exposure to the atmosphere [136]. Additionally, it is well known that AgNPs can be oxidized and shed Ag^+^ ions in aqueous media, by reacting with dissolved O_2_ [22]. Storage of AgNPs in dispersion for several weeks was shown to considerably increase the toxicity of the AgNP dispersion toward human mesenchymal stem cells, compared to freshly synthesized dispersions of AgNPs, due to an increased concentration of Ag^+^ ions. Finally, the toxicity of AgNPs can be affected by sonication dispersion protocols [137]. During sonication a large amount of energy is applied to the AgNP dispersion to enable the rupture of larger AgNP aggregates. However, the majority of this energy is converted into thermal energy, raising the temperature and potentially promoting the dissolution of AgNPs [22]. Higher concentrations of Ag^+^ ions in AgNP dispersions prepared without cooling the samples over ice during sonication were measured by atomic adsorption spectroscopy (AAS). Sonicated AgNP dispersions were more toxic to A549 epithelial cells than when prepared with cooling [138].

### 4.2. Transformation in the Cell Culture Media

**Figure 2:**
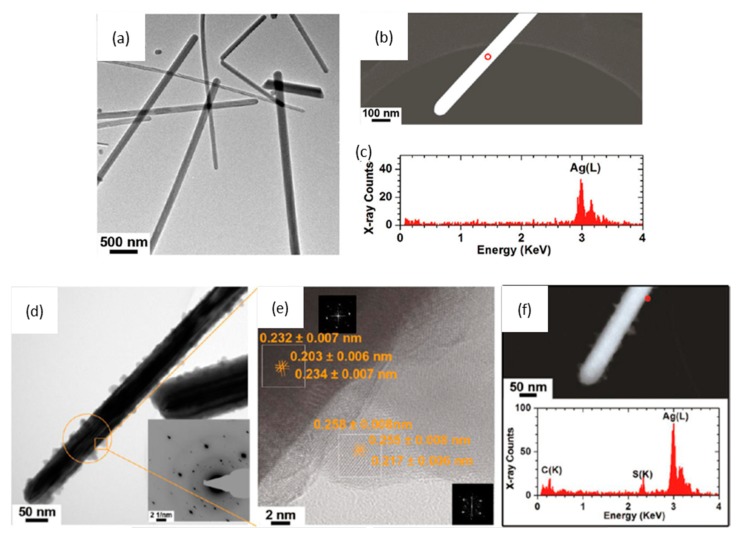

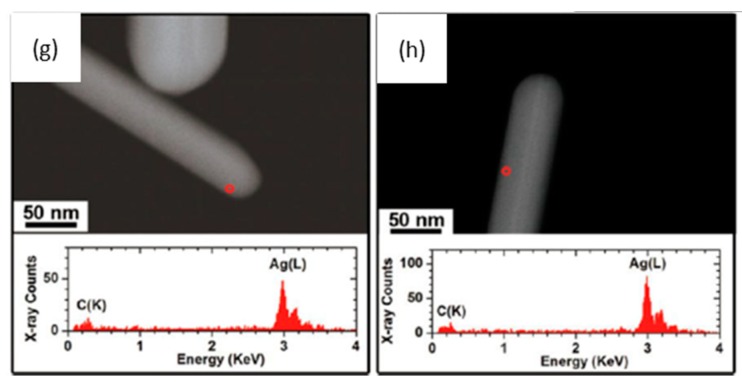
(**a**,**b**) Low-resolution bright field transmission electron microscopy (BF-TEM) image (**a**) and high angle annular dark field scanning transmission electron microscopy (HAADF-STEM) image (**b**) of as-synthesized silver nanowires (AgNWs); (**c**) The corresponding energy dispersive X-ray (EDX) spectrum collected from the area circled in (**b**); The two peaks at 2.98 and 3.15 keV correspond to the Ag(L_α_) and Ag(L_β_) peaks, respectively; (**d**–**h**) Physicochemical characterization of AgNWs incubated in various cell culture media for 1 h at 37 °C; (**d**–**f**) a high protein serum-free medium (DCCM-1, Biological Industries, Israel); (**g**) Dulbecco’s Modified Eagle Medium (DMEM); and (**h**) Roswell Park Memorial Institute (RPMI-1640) medium; (**d**) Representative BF-TEM image of AgNWs incubated in DCCM-1 medium, showing the formation of crystallites on the surface of the AgNWs. The inset is a selected area electron diffraction (SAED) pattern taken from the circled area (aperture size ~130 nm); (**e**) HRTEM image collected from the boxed area in (**d**) reveals that the crystallites have a different crystal structure than the original AgNWs. The lattice spacings of the crystals formed at the surface of the AgNWs (~0.26 and 0.22 nm) correspond to the (112) and (031) lattice spacings of Ag_2_S, respectively. The lattice spacings of the core of the nanowires (~0.23 and 0.20 nm) correspond to the interplanar spacings of metallic Ag. Insets are fast Fourier transform (FFT) patterns taken from the two-boxed areas; (**f**) HAADF-STEM image (top) taken from the same area as (**d**) and STEM-EDX spectra collected from the circled area (bottom), confirming the formation of Ag_2_S; (**g**,**h**) HAADF-STEM images (top) and EDX spectra (bottom) of AgNWs incubated in DMEM (**g**) and RPMI-1640 (**h**) cell media, indicating that AgNWs do not sulphidise in DMEM or RPMI-1640. Adapted with permission from [143]. Copyright 2013 American Chemical Society.

**Figure 3:**
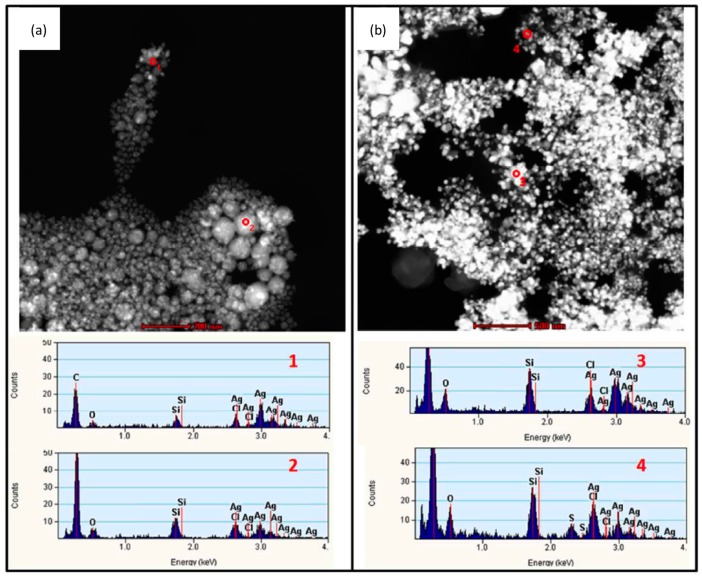

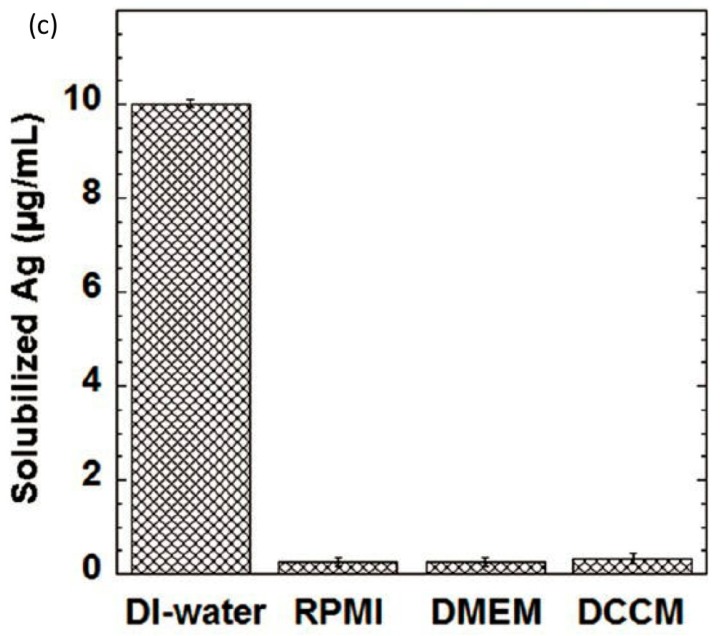
(**a**,**b**) HAADF-STEM-EDX characterization of the precipitates formed after the incubation of 17.0 µg/mL AgNO_3_ (equal to an Ag concentration of 10 μg/mL) in (**a**) RPMI-1640 and (**b**) DCCM-1 cell culture medium, at 37 °C for 0.5 h. The corresponding STEM-EDX spectra 1–4 were collected from the areas 1–4 marked in (**a**,**b**); The precipitates were collected by filtering the solution through 2 kDa filter membrane and were washed three times with DI-water. In both cell culture media, the particles have sizes that range from ~20 to ~200 nm. STEM-EDX analysis reveals that the precipitates in RPMI-1640 are probably a mixture of silver oxide and silver chloride. The insoluble compounds formed in DCCM-1 likely also contain silver sulphide; (**c**) Inductively coupled plasma optical emission spectroscopy (ICP-OES) analysis of solubilized silver concentrations of 17 μg/mL AgNO_3_ (equal to an Ag concentration of 10 μg/mL) in deionized (DI) water, RPMI-1640, DMEM, and DCCM-1 solutions, incubated at 37 °C for 0.5 h (*n* = 3). Although 100% of the free Ag^+^ ions were detected in the AgNO_3_ DI-water solution, the amount of solubilized silver in cell culture media was less than 0.5 μg/mL after incubation. The dissolved Ag^+^ ions are probably sequestered as insoluble precipitates in the cell media, indicating the limitation of using ICP-OES to study the kinetics of Ag^+^ dissolution in cell culture media. Adapted with permission from [143]. Copyright 2013 American Chemical Society.

To allow interaction with cells in culture, tissue or organisms, as-synthesized AgNPs are usually suspended in biocompatible aqueous tissue culture medium. Depending on the composition and exposure time to the biological environment, agglomeration/aggregation of the particles could take place or there may be alteration of their dissolution profile. Several factors such as ionic strength and composition of the dispersion medium [27,28,29,139,140], pH [22,141], dissolved organic matter [142], dissolved oxygen concentration [14] and temperature [26] have been shown to affect the stability of AgNPs and are consequently expected to have an impact on their bioreactivity. Therefore an assessment of the properties of AgNPs in the extracellular medium, before or during cell exposure tests, is also crucial. This has recently been highlighted by Chen *et al.*, who reported cell culture media-induced changes to the chemistry of silver nanowires (AgNWs) using high resolution analytical electron microscopy (HRTEM) (Figure 2) [143]. It was demonstrated that silver sulphide (Ag_2_S) crystals formed on the surface of AgNWs following incubation in DCCM-1 cell culture medium. Ionic silver released from the surface of AgNWs will transform to Ag_2_S due to its extremely low water solubility (*K*_sp_ = 5.92 × 10^−51^) [144]. This silver-to-silver sulphide transformation will substantially reduce the Ag^+^ ion release rate; therefore reduced AgNP toxicity could be expected. Indeed, some studies have shown low toxicity of Ag_2_S NPs and reduced antibacterial activity of AgNPs because of sulphidation [145,146]. Moreover, studies to determine the amount of Ag^+^ ions dissolved in media, which are known as one of the major factors affecting Ag toxicity, and the impact of this ionic species on cells are quite rare [27,28]. Inductively coupled plasma sources (ICP-OES and ICP-MS) and atomic absorption spectroscopy (AAS) techniques are commonly used to correlate the dissolution rate of AgNPs with their toxicity profiles. However, there are discrepancies in the data provided by different authors, probably due to the use of inconsistent methodologies (e.g., experimental setup, separation method) and incomplete NP characterization [24,60,112,147]. Moreover, AgNO_3_ is frequently used as a control to study the effects of free Ag^+^ ions but the fact that addition of AgNO_3_ to cell culture media rapidly leads to the precipitation of insoluble Ag compounds has not been addressed. Using analytical TEM techniques, we have shown that the precipitates from AgNO_3_ in RPMI-1640 medium consisted of small particles, with sizes from 20 to 200 nm, that were likely a mixture of silver oxide (*K*_sp_= 4 × 10^−11^) and silver chloride (*K*_sp_= 1.77 × 10^−10^) (Figure 3). The amount of free Ag^+^ measured by ICP-OES was less than 5% of the total Ag^+^ added [143]. Therefore, a combination of characterization techniques is necessary in order to understand which particulate species actually interact with the cells.

On the other hand, the addition of proteins in the cell culture medium could alter what the cell “sees” [148]. It is known that NPs are covered with serum proteins after their dispersion in serum-containing physiological media [149], leading to a so-called protein corona [150]. These proteins may be responsible for modifying the aggregation state of AgNPs and may even directly affect the degree of Ag^+^ ions released from the NP surface [151]. For instance, Kittler *et al.*, reported that 50 nm PVP-capped AgNPs suspended in RPMI medium containing bovine serum albumin (BSA) agglomerated, but the same particles remained dispersed if the BSA was replaced by foetal calf serum (FCS) [152]. The authors suggested that the albumin lipoprotein, glyco-protein and globulin content of FCS may have been responsible for a steric stabilization of the AgNPs. Yen *et al.*, linked variations in serum protein attachment to the surface of AgNPs and AuNPs to differences in their uptake mechanism and toxicity to macrophages. They suggested that negatively charged AuNPs (ζ potential from −56.64 to −78.81 mV) were able to adsorb serum proteins and enter cells *via* the phagocytic as well as the pinocytic pathway. However positively charged AgNPs ζ potential from 5.35 to 15.43 mV) could not adsorb serum proteins, which reduced uptake and exerted a lower cytotoxicity than AuNPs [153]. In another published study, BSA was found to interact strongly only with uncapped AgNPs compared to PVP- and citrate-capped AgNPs of similar sizes (surface charge not reported). The presence of serum albumin led to the aggregation of uncapped AgNPs and consequently lowered their antibacterial activity compared to the capped AgNPs [154].

There are also conflicting data in the literature about whether sulfur containing proteins such as albumin are able to alter the chemistry of AgNPs. In our own work we showed that PVP-capped AgNWs do not sulphidise in phosphate buffered saline (PBS) containing 5% cysteine or 5% BSA, but do sulphidise in the inorganic fraction of a serum-free cell culture medium (DCCM-1) [143]. Other reports have shown that Ag^+^ ions can bind strongly to both inorganic sulfur groups as well as organosulfur compounds, with the highest affinity for thiols, such as cysteine [155]. Ag^+^ ions may have a high affinity for thiols and cysteine, changing their biological activity; however, Ag^+^ ions may not be able to remove sulfur from biological molecules to form an inorganic sulphide without the existence of other oxidizing species.

### 4.3. Transformation in Pulmonary Surfactant

Pulmonary surfactant covers the entire alveolar region, decreasing the surface tension in the alveoli to prevent alveolar collapse. Human surfactant consists of about 80% phospholipids, 8% neutral lipids (cholesterol, triacylglycerol and free fatty acids) and 10% surfactant-specific proteins. The most abundant component of surfactant is phosphatidylcholine (PC), which accounts for 70%–80% of the total amount of lipids. Approximately 50% of PC is saturated in the dipalmitoylated form (DPPC) [156]. Furthermore, four surfactant-associated proteins have been described: The hydrophilic SP-A and SP-D and the hydrophobic SP-B and SP-C [157]. SP-A and SP-D are collectins, which play a fundamental role in host defence by facilitating phagocytosis of various bacterial and viral pathogens. On the other hand, SP-B and SP-C promote the rapid adsorption of the surfactant phospholipids at the air-liquid interface and maintain the stability of the lung system by influencing the molecular ordering of the phospholipid layer [156,158].

**Figure 4:**
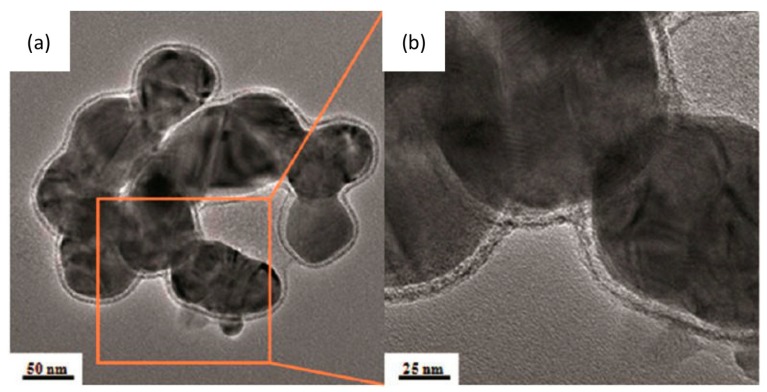

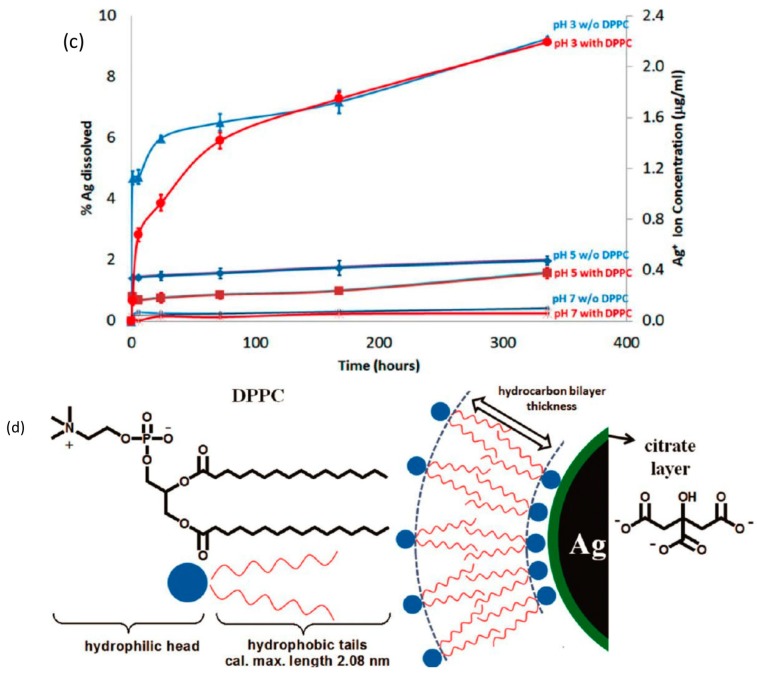
(**a**,**b**) BF-TEM images of 20 nm citrate-coated AgNPs incubated in a perchlorate pH 3 solution, in the presence of DPPC, for one day. Incubation of AgNPs in solutions containing DPPC resulted to a significant increase of the amorphous layer thickness around the particles, which implies the formation of DPPC layer(s) on the surface of AgNPs. The samples were negatively stained with uranyl acetate to enhance the contrast of lipid coating. The high affinity of electron dense uranyl ions to the carboxyl groups of citrate and the phosphate groups of DPPC results in dark contrast. Therefore, the outside layer of AgNPs, showing dark contrast, is likely to be the polar groups of DPPC facing the aqueous environment, whereas the hydrophilic heads of the inner layer of DPPC likely interact with the citrate layer; (**c**) Ag^+^ ion release from AgNPs incubated in perchlorate solutions at pH 3, 5 or 7, in the presence and absence of DPPC. The DPPC coating of AgNPs may serve as a semipermeable layer, delaying the release of Ag^+^ ions but without significantly altering the total amount of Ag^+^ released after two weeks; (**d**) A schematic illustration of a model of DPPC bilayer structure on the surface of AgNPs. The DPPC surfactant molecule consists of a trimethyl ammonium bounded to an acidic phosphate, providing a hydrophilic zwitterionic headgroup and two hydrophobic fatty acid tails comprised by 16 hydrocarbons. There is likely a formation of a lipid bilayer structure on the surface of citrate coated AgNPs, with hydrophobic tails associating with each other, whereas the hydrophilic groups are oriented towards the aqueous environment and the citrate layer, respectively. Adapted with permission from [23]. Copyright 2013 American Chemical Society.

Possible transformations of the AgNPs when they come into contact with the lung surfactant have to be addressed because any interactions could both disrupt the physiological surfactant function, as well as altering their subsequent cellular effects. So far, only a few studies have investigated the stability of AgNPs in environments fully representative of the lung. For example, Stebounova *et al.*, studied the stability of two types of AgNPs, PVP-coated and polymer-coated, in artificial interstitial and lysosomal fluids [28]. Their experimental results and the extended Derjaguin-Landau-Verwey-Overbeek (DLVO) model calculations showed that PVP-capped AgNPs precipitated but polymer-coated AgNPs, with a higher negative surface charge, were more stable in the simulated fluids. However, these simulated fluids were lacking any surfactant components. A few other studies have examined the colloidal stabilization of other types of NPs in rat bronchoalveolar lavage (BAL) [159], phosphate buffered saline (PBS) containing bovine serum albumin (BSA) and DPPC [160,161] or semisynthetic lung fluids consisting of DPPC, palmitoyl-oleoyl-phosphatidylglycerol and SP-B (70:30:1 *w*/*w*/*w*) [162]. These studies found that NPs were well-dispersed in the presence of DPPC and proteins and that synthetic lung fluid was as effective as BAL in dispersing the NPs. Recently, the stability of citrate-capped AgNPs in DPPC was investigated as a function of pH (Figure 4) [23]. A decrease in pH was found to accelerate the kinetics of Ag^+^ ion release but also promoted particle aggregation and coarsening. DPPC however, delayed the release of Ag^+^ ions, without significantly altering the total amount of Ag^+^ released after two weeks. By coating the AgNPs, DPPC improved their dispersion and inhibited aggregation and coarsening. This may allow AgNPs to enter cells more easily, leading to a greater ROS formation within cells, reduced cell viability and increased DNA damage.

The coating of AgNPs by phospholipids could have serious implications on pulmonary homeostasis by interfering with biophysical surfactant function. One study, for example, investigated the effect of gold nanoparticles (AuNPs) as a model metal air pollutant on the surfactant function of a semisynthetic surfactant, composed of DPPC, palmitoyl-oleoyl-phosphatidylglycerol (POPG), and SP-B (70:30:1 *w*/*w*/*w*) [162]. AuNPs markedly inhibited the adsorption of pulmonary surfactant at the air-liquid interface, induced a dysfunction during film compression and inhibited the re-spreading during film expansion. Since AuNPs were shown to be coated by phospholipids, the authors speculated that these coated particles could adsorb at the air-liquid interface and thereby inhibit the adsorption of free phospholipids. Furthermore, how the binding of surfactant components on AgNPs may affect their toxicity is not known. Research on other types of nanomaterials has shown, for instance, that a bovine surfactant preparation (Survanta) and SP-A increased the uptake of TiO_2_ particles into primary rat alveolar macrophages [163]. Konduru *et al.*, showed that single walled carbon nanotubes (SWCNTs) could bind phosphatidylserine (PS), which facilitated their recognition and internalization by several types of phagocytic cells, such as mouse RAW264.7 macrophages, primary monocyte-derived human macrophages and dendritic cells and primary rat brain microglia [164]. Bound surfactant components could also affect the intracellular response to AgNPs. Although there are currently no data on AgNPs, studies on other systems have emphasised the importance of the NP-surfactant interactions. For example, recent findings demonstrated that pre-coating multi-walled carbon nanotubes (MWCNTs) with Curosurf^®^ evoked an increase in ROS, inflammatory chemokine release and apoptosis in human monocyte derived macrophages [165]. An increased ROS production by normal human primary bronchial epithelial cells and A549 type II alveolar epithelial cells was also reported for single-walled carbon nanotubes (SWCNTs) dispersed in DPPC [166]. Moreover, coating various types of NPs with the surfactant-associated proteins SP-A and SP-D, led to the agglomeration of the particles [97,98]. The selective binding of SP-A and SP-D to carbon nanotubes [99], the adsorption of SP-A on metal oxide NPs [97] and the adsorption of SP-D on AuNPs [98] has already been shown. Recently, Ruge *et al.*, revealed a pronounced binding of SP-A to hydrophobic magnetite nanoparticles (mNPs) whereas SP-D preferentially adsorbed on hydrophilic mNPs [100]. These findings stress the need to further understand possible interactions between AgNPs and components of the lung surfactant and how these could affect cellular uptake, clearance, translocation and toxic effects in the lung.

### 4.4. Internalization of AgNPs by Cells and Transformation Inside the Cell

Uptake, internalization and transformation of AgNPs inside cells can be used to predict their potential bioreactivity. The pathways by which NPs enter cells can vary based on the cell type, chemical composition of the NP surface [153], particle size and agglomeration state [117]. Endocytosis is a form of active transport in which cells take up objects by enclosing them in vesicles or vacuoles pinched off from their cytoplasmic membrane [167]. The endocytotic processes that enclose NPs in membrane vesicles include phagocytosis, pinocytosis, and caveolae-dependent or clathrin-mediated endocytosis [168,169,170,171]. Phagocytosis is typically restricted to specialized mammalian cells, like macrophages, and involves the ingestion of large particles by large vesicles called phagosomes (diameter > 250 nm) [172]. Smaller particles, ranging from a few up to hundreds of nanometres are internalized by pinocytosis or macropinocytosis, which occurs in almost all cell types. Energy-dependent clathrin-mediated endocytosis is probably the primary characterized mechanism for the cellular uptake of NPs [173]. Greulich *et al.*, investigated the uptake of 50 nm PVP-coated AgNPs by human mesenchymal stem cells and their intracellular distribution [174]. Their results demonstrated that clathrin-mediated endocytosis and macropinocytosis were the primary uptake mechanisms. In another study, Haase *et al.*, suggested that THP-1 macrophages may internalize AgNPs through both phagocytic and non-phagocytic mechanisms [117]. TEM imaging revealed aggregated AgNPs in vesicles but also individual particles distributed throughout the cytoplasm without being surrounded by membrane envelopes. The authors speculated that aggregated AgNPs are incorporated *via* phagocytosis while single NPs enter cells *via* non-phagocytic routes. Other authors have also suggested that NPs are able to enter cells by direct penetration of the cell membrane *via* diffusion, membrane fluidity, passing through ion-channels or by adhesive interactions (electrostatic forces, Van der Waals- or steric interactions) [175,176], but these mechanisms are controversial. Moreover, there is currently limited understanding on the cellular fate of NPs after they enter cells. After endocytosis, NPs should normally reside in membrane-bound vesicles [177,178], *i.e.*, endosomes that would later evolve into lysosomes or autophagosomes. Some studies, however, also report the presence of NPs in the cytosol [128], in mitochondria [179], and in the nucleus [15,117,118]. The mechanisms through which NPs can escape from endosomes are not understood, but seem to depend on the core composition [180], surface composition [181], charge [182] and shape of the NPs [183,184].

The surface charge of NPs can largely influence the uptake mechanism by cells. In general, due to the negatively charged character of the cell plasma membrane, cationic NPs are internalized more efficiently than neutral or anionic NPs [185]. Macropinocytosis seems to be the dominant mechanism for the uptake of positively charged NPs, while a clathrin-and caveolae-independent endocytosis may mainly contribute to the uptake of negatively charged NPs [186]. For example, bare AuNPs (positively charged) were taken up by macropinocytosis and clathrin- and caveolin-mediated endocytosis, whereas PEG-coated AuNPs (negatively charged) mostly entered cells by caveolin- and/or clathrin-mediated endocytosis, but not by macropinocytosis [187]. The shape of nanomaterials is another important factor that can determine their uptake mechanism. This can have important implications on the long-term consequences of nanomaterial exposure; for example since frustrated phagocytosis is considered as an important factor in the initiation of an inflammatory response due to exposure to fibres with a high aspect ratio. Comparisons with asbestos fibres in the lung and the induction of mesothelioma have been made to both AgNWs and multi-walled carbon nanotubes with high aspect ratios [188,189]. In a recent study, backscatter scanning electron microscopy (BSE) was used to investigate the cut-off length for the frustrated phagocytosis of AgNWs *in vitro* and *in vivo* [21]. While *in vitro* frustrated phagocytosis by THP-1 macrophages could be observed with fibres ≥14 μm, *in vivo* studies showed incomplete uptake at a fibre length of ≥10 μm. The same group had shown that inflammation in the pleural space after intrapleural injection of AgNWs in mice occurred at a length ≥5 μm [189]. Therefore, the onset of inflammation could not be correlated with the onset of frustrated phagocytosis. However, the limited resolution of the technique used did not allow for any quantitative analysis of dissolution or changes to the AgNP morphology and chemistry after cellular uptake.

The amount and rate of uptake of AgNPs by the cells can differ by several orders of magnitude depending on the size, shape, surface charge, and surface functionalization of the particles [190]. Analytical techniques such as ICP-MS, ICP-OES or AAS are often used to quantify the amount of AgNPs taken up by cells. However, attention should be paid to the protocol used for the digestion of tissue samples, since some procedures commonly used can lead to low Ag recoveries due to AgCl precipitation [191]. Furthermore, the use of these techniques does not efficiently distinguish between AgNPs attached to the cellular membrane and those internalized by the cells. To overcome this limitation, AgNPs labelled with a fluorescein derivative (DTAF) were used to quantify cellular uptake by macrophages (RAW 264.7) after 3 h exposure. TEM and confocal microscopy were used to confirm the localization of AgNPs in the cellular cytoplasm [192]. Confocal microscopy was also employed by Cronholm *et al.*, who detected AgNPs in the cytosol in 80% of the investigated cells [118]. When developing a methodology based on fluorescently-labelled NPs, however, it is important to keep in mind that the attachment of a fluorochrome to the NP surface may alter the physicochemical properties of AgNPs, thus influencing their cellular uptake, or the label may detach from the NPs inside cells, leading to artefacts. Pratsinis *et al.*, employed dark-field microscopy to visualize the intracellular localization of AgNPs of different sizes [193]. Both 5.7 and 16.8 nm AgNPs were efficiently taken up by mouse macrophages after 24 h exposure. Their results also indicated that NP agglomeration had taken place following cell internalization. In another study, macrophages were exposed to 5 and 100 nm PVP-coated AgNPs [110]. Using TEM and optical ultra-resolution imaging, a lower amount of 5 nm PVP-coated AgNPs was detected inside macrophage cells compared to 100 nm particles. However, a higher cytotoxicity was induced by the smaller sized AgNPs (LD_50_ was 6.25 µg/mL for 5 nm AgNPs and 25 µg/mL for 100 nm AgNPs). The authors suggested that the techniques used didn’t provide the spatial resolution needed to visualize the 5 nm AgNPs inside cells. However, additional data on the solubility of AgNPs would be necessary in order to confirm whether the observed cytotoxicity was due to higher dissolution, as would be expected due to the increased surface area to volume ratio.

**Figure 5:**
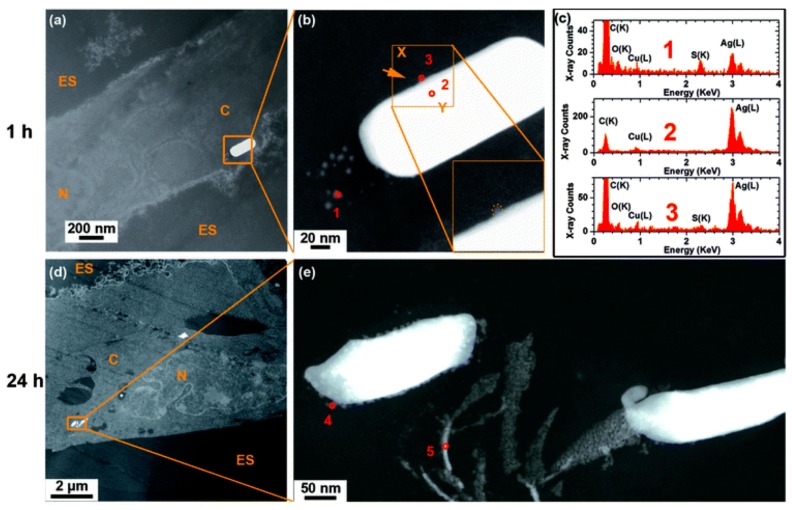

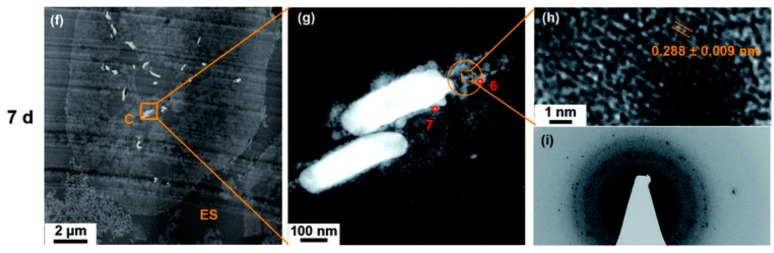
Changes in the morphology and chemistry of AgNWs as a function of time, after their uptake by TT1 epithelial cells at 1 h (**a**–**c**), 24 h (**d**,**e**) and seven days (**f**–**i**), following a pulsed exposure, using unstained cell sections. (ES = extracellular space; C = cytoplasm; N = nucleus) (**a**) HAADF-STEM image of an AgNW inside the cell cytoplasm showing particles surrounding the tip of the AgNW; (**b**) A corresponding higher resolution HAADF-STEM image depicts the boxed area in (**a**); The insert in (**b**) shows a higher magnification image of the AgNW edge; (**c**) STEM-EDX spectra taken from the corresponding areas 1–3 marked in (**b**); Ag(L) peaks and S(K) peaks were detected in the STEM-EDX spectra of these small particles, indicating the formation of Ag_2_S (**d**,**e**) HAADF-STEM images, where image (**e**) depicts the boxed area in (**d**), showing that dissolution and sulphidation of the AgNWs was more substantial after 24 h (**f**–**i**) HAADF-STEM images, where (**g**) depicts the boxed area in (**f**); (**h**) An HRTEM image of the boxed area in image (**g**). The particles had a lattice spacing of 0.29 nm, close to the mono-clinic structure of Ag_2_S (-112) (**i**) SAED pattern from the circled area in (**g**), using a selected area aperture size of ~130 nm. The interplanar spacings measured from the SAED patterns were consistent with bulk monoclinic Ag_2_S. Adapted with permission from [123]. Copyright 2013 The Royal Society of Chemistry.

In order to visualize the distribution and chemistry of AgNPs inside cells, a combination of electron microscopy techniques (TEM, SEM and dual beam FIB combined with EDX analysis) has been applied in a number of studies. In a study by Park *et al.*, no presence of AgNPs was detected in damaged cells, suggesting that the acute toxicity of these NPs was due to their intracellular dissolution [194]. Vanwinkle *et al.*, observed an intracellular size reduction of AgNPs in a rat type I-like alveolar epithelial cell line by TEM [195]. On the contrary, Carlson *et al.*, concluded that 55 nm AgNPs (unspecified “hydrocarbon” coating) did not dissolve after uptake by a rat alveolar macrophage cell line [106]. TEM imaging showed that AgNPs remained at the same size, even when they were inside cellular compartments responsible for digestive processes, such as vacuoles. In another study, 20–40 nm AgNPs were found as agglomerates inside A549 lung cells [118]. Moreover, a change in particle morphology was observed by TEM, as smaller particles had formed close to the original AgNPs inside cells. The authors suggested that these small particles could have been formed due to the precipitation of silver salts/complexes following release of Ag^+^ ions intracellularly, or resulting from a re-nucleation of AgNPs. Since no chemical analysis of these smaller particles was performed, however, these hypotheses could not be tested. In our recent work, we employed high resolution analytical TEM techniques to elucidate the cellular uptake and reactivity of AgNWs inside a human alveolar epithelial type 1-like cell line (Figure 5) [123]. AgNWs were found in the cytoplasm and membrane-bound vesicles by bright field TEM (BF-TEM). However, we observed that staining agents, such as osmium tetroxide and potassium ferricyanide, which are frequently used during biological sample preparation for TEM (to enhance the electron density of cellular compartments and organelles), caused substantial changes to the morphology and chemistry of AgNWs. Therefore, we eliminated heavy metal staining processes and took precautions to avoid AgNW sulphidation in ambient air and in the cell culture medium [143]. High angle annular dark field (HAADF) scanning transmission electron microscopy (STEM), a technique which is highly sensitive to local variations in the atomic number within a sample, and energy-dispersive X-ray spectroscopy (EDX) were used to observe the morphological and chemical features of individual AgNWs within TT1 cells. These techniques provided compelling evidence that Ag^+^ is released from AgNWs inside epithelial cells and that Ag^+^ ions subsequently precipitate as Ag_2_S. We suggested that this process occurred by the action of sulphide species including H_2_S, HS^−^ and S^2−^ inside cells. Cell viability studies showed no evidence of cytotoxicity and reactive oxygen species were not observed on exposure of cells to AgNWs. We therefore proposed that Ag_2_S formation reduces the amount of bioavailable free Ag^+^, significantly limiting short-term toxicological effects. These findings underline the importance of careful experimental design and sample preparation and the use of a combination of characterization techniques in order to understand transformations of AgNPs inside cells, and the consequences on the bioreactivity of the particles. One of the main challenges is the ability to measure the amount of “free” ionic and particulate Ag in the biological *milieu*. The use of ICP-MS to measure the concentration of Ag^+^ ions or AgNPs within cells [60] is limited by the fact that ICP-MS detects the total metal content without distinguishing between metal oxidation states. Therefore, the development of methods for the quantification of oxidation rates of AgNPs inside cells is crucial for future research. In our group, fluorescent dyes and confocal microscopy were employed to visualize the amount of ionic Zn^2+^ released from ZnO NWs inside human macrophages [196]. The development of a sensitive and selective colorimetric Ag^+^ detection method, however, is still under research. Application of similar methods would provide valuable insight into the mechanisms by which AgNPs exert their biological effects and help de-convolute the effects of Ag^+^ ions and Ag particles.

## 5. Conclusions

The available literature data strongly suggest that silver nanomaterials are a potential health hazard. Therefore, their increased production and use in consumer products must be accompanied by appropriate risk assessment processes and the design of regulatory frameworks that protect public health. *In vivo* studies have revealed the inflammatory potential of AgNPs in the lungs and that prolonged exposures could lead to the development of pulmonary function abnormalities. Following inhalation, AgNPs were discovered to translocate to the blood circulation and were subsequently distributed throughout the main organs. Meanwhile, evidence of genotoxicity has also been reported. Moreover, the toxicity of AgNPs has been demonstrated *in vitro* for several different types of alveolar cells (e.g., epithelial and macrophages). The toxicological outcomes upon exposure to Ag nanomaterials include oxidative stress, lipid peroxidation, inhibition of mitochondrial activity, damage to DNA, and cell apoptosis. However, a predictive toxicological paradigm for AgNP toxicity, which translates their *in vitro* cellular effects to their *in vivo* toxicological potential, has not been successfully established. Several discrepancies still exist between the published results, in part due to the lack of sufficient controls over the cellular systems investigated and the particles tested. This highlights the need to develop valid *in vitro* systems and standardized inter-laboratory or international methods in the future. The use of accurate characterization techniques and the development of advanced nanometrology protocols, to analyse the nanomaterials in their near native state in the lung, in particular dynamic events, will be crucial in order to elucidate the mechanisms of biological action of inhaled silver nanomaterials.

To achieve this, advances in several areas need to be made. First, there is a lack of data on the magnitude of exposure to AgNPs both in the workplace and during use of consumer products containing AgNPs. The environmental concentrations of AgNPs will have to be quantified, in order to inform the selection of appropriate doses in toxicological studies. In the past, unrealistically high doses have been used in an effort to determine the mechanisms of biological action of AgNPs. Accurate dosimetry in laboratory studies of the effects of inhaled particles has been, and still is, a subject of concern. For instance, a predictive NP deposition model has shown that the amount of inhaled NPs depositing per unit surface area of the alveolar region was several orders of magnitude lower than doses routinely used in *in vitro* studies with alveolar epithelial cells [190]. Moreover, viable *in vitro* alternatives to *in vivo* testing need to be developed, that will provide a holistic understanding of the “bionano” systems. The selection of cell types to be tested is crucial in this case, since the toxic effects of AgNPs clearly depend on the type of cell encountered. Approaches relying on multiple cell co-cultures and experimental setups that can be used at the air-liquid interface need to be further developed, to represent realistic exposure scenarios of AgNP inhalation. Moreover, these systems will have to integrate surfactant interactions, in order to accurately mimic the *in vivo* situation.

Furthermore, to elucidate the mechanisms of biological action of AgNPs, characterization of the particles at all stages of the *in vitro* experiment is crucial. AgNPs are highly dynamic systems and their properties can dramatically change when incubated in biological media. Examining the effects of cell culture media (ionic salts and proteins) and the lung surfactant (lipids and proteins) on the physicochemistry of AgNPs is essential in order to understand the characteristics of the system that the cells encounter. Experimental techniques commonly used for the characterization of AgNPs in the past have to be scrutinized, since many of them have been shown to possess limitations. Accurate characterization of the particles should rely on several complementary techniques across biological and physical sciences disciplines. In this area, cryo- and high resolution analytical transmission electron microscopy techniques have been shown to be extremely useful, providing spatially resolved information on the morphology and chemistry of AgNPs.

Finally, characterization of AgNPs should also take place at the particle-cell interface. Investigating possible transformations of the particles within the cellular environment can provide valuable information about the interactions of AgNPs with cellular components and the mode of their biological action. For instance, there is no agreed consensus whether bioreactivity is exerted by AgNPs themselves, by Ag^+^ ions released from their surface or by a synergistic effect between AgNPs and Ag^+^ ions. Novel approaches based on the correlative application of high spatial and energy resolution analytical microscopy techniques, and the development of new metrology methods to quantify the amount of intracellular Ag^+^ ions, may offer an improved understanding of the mechanisms by which AgNPs interact with cells. Understanding which properties of AgNPs drive their bioreactivity will guide the regulation of these materials, provide guidelines for their safe handling and enable the design of safe consumer products.

## References

[1] Chen X., Schluesener H.J. (2008). Nanosilver: A nanoproduct in medical application. Toxicol. Lett..

[2] Marambio-Jones C., Hoek E.M.V. (2010). A review of the antibacterial effects of silver nanomaterials and potential implications for human health and the environment. J. Nanopart. Res..

[3] Hansen S.F., Baun A. (2012). When enough is enough. Nat. Nanotechnol..

[4] Wijnhoven S., Peijnenburg W., Herberts C.A., Hagens W.I., Oomen A.G., Heugens E., Roszek B., Bisschops J., Gosens I., van de Meent D. (2009). Nano-silver: A review of available data and knowledge gaps in human and environmental risk assessment. Nanotoxicology.

[5] Link S., el-Sayed M.A. (1999). Spectral properties and relaxation dynamics of surface plasmon electronic oscillations in gold and silver nanodots and nanorods. J. Phys. Chem. B.

[6] Kamat P.V. (2002). Photophysical, photochemical and photocatalytic aspects of metal nanoparticles. J. Phys. Chem. B.

[7] The Project on Emerging Nanotechnologies. http://www.nanotechproject.org/cpi/.

[8] Eturska M., Obreshkova E. (1979). Argyria in the prolonged use of adsorgan. Vutreshni Boles..

[9] Spencer W.H., Garron L.K., Contreras F., Hayes T.L., Lai C. (1980). Endogenous and exogenous ocular and systemic silver deposition. Trans. Ophthalmol. Soc. U. K..

[10] Fabrega J., Luoma S.N., Tyler C.R., Galloway T.S., Lead J.R. (2011). Silver nanoparticles: Behaviour and effects in the aquatic environment. Environ. Int..

[11] Foldbjerg R., Dang D.A., Autrup H. (2011). Cytotoxicity and genotoxicity of silver nanoparticles in the human lung cancer cell line, A549. Arch. Toxicol..

[12] Park M.V., Neigh A.M., Vermeulen J.P., de la Fonteyne L.J., Verharen H.W., Briede J.J., van Loveren H., de Jong W.H. (2011). The effect of particle size on the cytotoxicity, inflammation, developmental toxicity and genotoxicity of silver nanoparticles. Biomaterials.

[13] Costa C.S., Ronconi J.V., Daufenbach J.F., Goncalves C.L., Rezin G.T., Streck E.L., Paula M.M. (2010). *In vitro* effects of silver nanoparticles on the mitochondrial respiratory chain. Mol. Cell. Biochem..

[14] Suresh A.K., Pelletier D.A., Wang W., Morrell-Falvey J.L., Gu B., Doktycz M.J. (2012). Cytotoxicity induced by engineered silver nanocrystallites is dependent on surface coatings and cell types. Langmuir.

[15] Kim H.R., Kim M.J., Lee S.Y., Oh S.M., Chung K.H. (2011). Genotoxic effects of silver nanoparticles stimulated by oxidative stress in human normal bronchial epithelial (BEAS-2B) cells. Mutat. Res..

[16] Stoehr L.C., Gonzalez E., Stampfl A., Casals E., Duschl A., Puntes V., Oostingh G.J. (2011). Shape matters: Effects of silver nanospheres and wires on human alveolar epithelial cells. Part. Fibre Toxicol..

[17] Arora S., Jain J., Rajwade J.M., Paknikar K.M. (2008). Cellular responses induced by silver nanoparticles: *In vitro* studies. Toxicol. Lett..

[18] Kang K., Jung H., Lim J.S. (2012). Cell death by polyvinylpyrrolidine-coated silver nanoparticles is mediated by ROS-dependent signaling. Biomol. Ther..

[19] Piao M.J., Kang K.A., Lee I.K., Kim H.S., Kim S., Choi J.Y., Choi J., Hyun J.W. (2011). Silver nanoparticles induce oxidative cell damage in human liver cells through inhibition of reduced glutathione and induction of mitochondria-involved apoptosis. Toxicol. Lett..

[20] AshaRani P.V., Mun G.L.K., Hande M.P., Valiyaveettil S. (2009). Cytotoxicity and genotoxicity of silver nanoparticles in human cells. ACS Nano.

[21] Schinwald A., Donaldson K. (2012). Use of back-scatter electron signals to visualise cell/nanowires interactions *in vitro* and *in vivo*; Frustrated phagocytosis of long fibres in macrophages and compartmentalisation in mesothelial cells *in vivo*. Part. Fibre Toxicol..

[22] Liu J.Y., Hurt R.H. (2010). Ion release kinetics and particle persistence in aqueous nano-silver colloids. Environ. Sci. Technol..

[23] Leo B.F., Chen S., Kyo Y., Herpoldt K.L., Terrill N.J., Dunlop I.E., McPhail D.S., Shaffer M.S., Schwander S., Gow A. (2013). The stability of silver nanoparticles in a model of pulmonary surfactant. Environ. Sci. Technol..

[24] Zhang W., Yao Y., Sullivan N., Chen Y. (2011). Modeling the primary size effects of citrate-coated silver nanoparticles on their ion release kinetics. Environ. Sci. Technol..

[25] Tejamaya M., Romer I., Merrifield R.C., Lead J.R. (2012). Stability of citrate, PVP, and PEG coated silver nanoparticles in ecotoxicology media. Environ. Sci. Technol..

[26] Kittler S., Greulich C., Diendorf J., Köller M., Epple M. (2010). Toxicity of silver nanoparticles increases during storage because of slow dissolution under release of silver ions. Chem. Mater..

[27] Yang X., Gondikas A.P., Marinakos S.M., Auffan M., Liu J., Hsu-Kim H., Meyer J.N. (2012). Mechanism of silver nanoparticle toxicity is dependent on dissolved silver and surface coating in caenorhabditis elegans. Environ. Sci. Technol..

[28] Stebounova L., Guio E., Grassian V. (2011). Silver nanoparticles in simulated biological media: A study of aggregation, sedimentation, and dissolution. J. Nanopart. Res..

[29] Liu J., Sonshine D.A., Shervani S., Hurt R.H. (2010). Controlled release of biologically active silver from nanosilver surfaces. ACS Nano.

[30] Xiu Z.M., Zhang Q.B., Puppala H.L., Colvin V.L., Alvarez P.J.J. (2012). Negligible particle-specific antibacterial activity of silver nanoparticles. Nano Lett..

[31] Navarro E., Piccapietra F., Wagner B., Marconi F., Kaegi R., Odzak N., Sigg L., Behra R. (2008). Toxicity of silver nanoparticles to *Chlamydomonas reinhardtii*. Environ. Sci. Technol..

[32] Choi O., Hu Z. (2008). Size dependent and reactive oxygen species related nanosilver toxicity to nitrifying bacteria. Environ. Sci. Technol..

[33] Fabrega J., Fawcett S.R., Renshaw J.C., Lead J.R. (2009). Silver nanoparticle impact on bacterial growth: Effect of pH, concentration, and organic matter. Environ. Sci. Technol..

[34] Yin L., Cheng Y., Espinasse B., Colman B.P., Auffan M., Wiesner M., Rose J., Liu J., Bernhardt E.S. (2011). More than the ions: The effects of silver nanoparticles on lolium multiflorum. Environ. Sci. Technol..

[35] Eckhardt S., Brunetto P.S., Gagnon J., Priebe M., Giese B., Fromm K.M. (2013). Nanobio silver: Its interactions with peptides and bacteria, and its uses in medicine. Chem. Rev..

[36] Kuhlbusch T.A., Asbach C., Fissan H., Gohler D., Stintz M. (2011). Nanoparticle exposure at nanotechnology workplaces: A review. Part. Fibre Toxicol..

[37] Sun T.Y., Gottschalk F., Hungerbuhler K., Nowack B. (2014). Comprehensive probabilistic modelling of environmental emissions of engineered nanomaterials. Environ. Pollut..

[38] Ostraat M.L., Thornburg J.W., Malloy Q.G.J. (2013). Measurement strategies of airborne nanomaterials. Environ. Eng. Sci..

[39] Park J., Kwak B.K., Bae E., Lee J., Kim Y., Choi K., Yi J. (2009). Characterization of exposure to silver nanoparticles in a manufacturing facility. J. Nanopart. Res..

[40] Tsai C.-J., Wu C.-H., Leu M.-L., Chen S.-C., Huang C.-Y., Tsai P.-J., Ko F.-H. (2009). Dustiness test of nanopowders using a standard rotating drum with a modified sampling train. J. Nanopart. Res..

[41] Nazarenko Y., Han T.W., Lioy P.J., Mainelis G. (2011). Potential for exposure to engineered nanoparticles from nanotechnology-based consumer spray products. J. Expos. Sci. Environ. Epidemiol..

[42] Lorenz C., Hagendorfer H., Goetz N., Kaegi R., Gehrig R., Ulrich A., Scheringer M., Hungerbühler K. (2011). Nanosized aerosols from consumer sprays: Experimental analysis and exposure modeling for four commercial products. J. Nanopart. Res..

[43] Mitrano D.M., Rimmele E., Wichser A., Erni R., Height M., Nowack B. (2014). Presence of nanoparticles in wash water from conventional silver and nano-silver textiles. ACS Nano.

[44] Oberdörster G., Oberdörster E., Oberdörster J. (2005). Nanotoxicology: An emerging discipline evolving from studies of ultrafine particles. Environ. Health Perspect..

[45] Bakand S., Hayes A., Dechsakulthorn F. (2012). Nanoparticles: A review of particle toxicology following inhalation exposure. Inhal. Toxicol..

[46] Alfaro-Moreno E., Nawrot T.S., Nemmar A., Nemery B. (2007). Particulate matter in the environment: Pulmonary and cardiovascular effects. Curr. Opin. Pulm. Med..

[47] Nel A., Xia T., Mädler L., Li N. (2006). Toxic potential of materials at the nanolevel. Science.

[48] Li J.J., Muralikrishnan S., Ng C.T., Yung L.Y., Bay B.H. (2010). Nanoparticle-induced pulmonary toxicity. Exp. Biol. Med..

[49] Tetley T.D. (2007). Health effects of nanomaterials. Biochem. Soc. Trans..

[50] Blank F., Gehr P., Rothen-Rutishauser B. (2009). *In vitro* human lung cell culture models to study the toxic potential of nanoparticles. Nanotoxicity.

[51] Lundborg M., Johard U., Lastbom L., Gerde P., Camner P. (2001). Human alveolar macrophage phagocytic function is impaired by aggregates of ultrafine carbon particles. Environ. Res..

[52] Muhlfeld C., Gehr P., Rothen-Rutishauser B. (2008). Translocation and cellular entering mechanisms of nanoparticles in the respiratory tract. Swiss Med. Wkly..

[53] Hillery A.M., Lloyd A.W., Swarbrick J. (2001). Drug Delivery and Targeting for Pharmacists and Pharmaceutical Scientist.

[54] Hussain S.M., Schlager J.J. (2009). Safety evaluation of silver nanoparticles: Inhalation model for chronic exposure. Toxicol. Sci..

[55] Takenaka S., Karg E., Roth C., Schulz H., Ziesenis A., Heinzmann U., Schramel P., Heyder J. (2001). Pulmonary and systemic distribution of inhaled ultrafine silver particles in rats. Environ. Health Perspect..

[56] Sung J.H., Ji J.H., Yoon J.U., Kim D.S., Song M.Y., Jeong J., Han B.S., Han J.H., Chung Y.H., Kim J. (2008). Lung function changes in sprague-dawley rats after prolonged inhalation exposure to silver nanoparticles. Inhal. Toxicol..

[57] Kim Y.S., Kim J.S., Cho H.S., Rha D.S., Kim J.M., Park J.D., Choi B.S., Lim R., Chang H.K., Chung Y.H. (2008). Twenty-eight-day oral toxicity, genotoxicity, and gender-related tissue distribution of silver nanoparticles in sprague-dawley rats. Inhal. Toxicol..

[58] Kim W.Y., Kim J., Park J.D., Ryu H.Y., Yu I.J. (2009). Histological study of gender differences in accumulation of silver nanoparticles in kidneys of fischer 344 rats. J. Toxicol. Environ. Health Part A.

[59] Tang J., Xiong L., Wang S., Wang J., Liu L., Li J., Yuan F., Xi T. (2009). Distribution, translocation and accumulation of silver nanoparticles in rats. J. Nanosci. Nanotechnol..

[60] Wang X., Ji Z., Chang C.H., Zhang H., Wang M., Liao Y.-P., Lin S., Meng H., Li R., Sun B. (2014). Use of coated silver nanoparticles to understand the relationship of particle dissolution and bioavailability to cell and lung toxicological potential. Small.

[61] Danscher G., Norgaard J.O., Baatrup E. (1987). Autometallography: Tissue metals demonstrated by a silver enhancement kit. Histochemistry.

[62] Ji J.H., Jung J.H., Kim S.S., Yoon J.U., Park J.D., Choi B.S., Chung Y.H., Kwon I.H., Jeong J., Han B.S. (2007). Twenty-eight-day inhalation toxicity study of silver nanoparticles in sprague-dawley rats. Inhal. Toxicol..

[63] Hyun J.-S., Lee B.S., Ryu H.Y., Sung J.H., Chung K.H., Yu I.J. (2008). Effects of repeated silver nanoparticles exposure on the histological structure and mucins of nasal respiratory mucosa in rats. Toxicol. Lett..

[64] Lee H.-Y., Choi Y.-J., Jung E.-J., Yin H.-Q., Kwon J.-T., Kim J.-E., Im H.-T., Cho M.-H., Kim J.-H., Kim H.-Y. (2010). Genomics-based screening of differentially expressed genes in the brains of mice exposed to silver nanoparticles via inhalation. J. Nanopart. Res..

[65] Song K.S., Sung J.H., Ji J.H., Lee J.H., Lee J.S., Ryu H.R., Lee J.K., Chung Y.H., Park H.M., Shin B.S. (2013). Recovery from silver-nanoparticle-exposure-induced lung inflammation and lung function changes in sprague dawley rats. Nanotoxicology.

[66] ACGIH (2001). Silver and compounds. Documentation of the Threshold Limit Values for Chemical Substances.

[67] Sung J.H., Ji J.H., Park J.D., Yoon J.U., Kim D.S., Jeon K.S., Song M.Y., Jeong J., Han B.S., Han J.H. (2009). Subchronic inhalation toxicity of silver nanoparticles. Toxicol. Sci..

[68] Mackison F.W., Partridge L.J., Stricoff R.S. (1981). NIOSH: Occupational Health Guidelines for Chemical Hazards: Silver Metal and Soluble Silver Compounds, National Institute for Occupational Safety and Health (NIOSH).

[69] Tsai S.-J., Ada E., Isaacs J., Ellenbecker M. (2009). Airborne nanoparticle exposures associated with the manual handling of nanoalumina and nanosilver in fume hoods. J. Nanopart. Res..

[70] Lee J.H., Kwon M., Ji J.H., Kang C.S., Ahn K.H., Han J.H., Yu I.J. (2011). Exposure assessment of workplaces manufacturing nanosized tio2 and silver. Inhal. Toxicol..

[71] Lee J., Ahn K., Kim S., Jeon K., Lee J., Yu I. (2012). Continuous 3-day exposure assessment of workplace manufacturing silver nanoparticles. J. Nanopart. Res..

[72] Kim J.S., Sung J.H., Ji J.H., Song K.S., Lee J.H., Kang C.S., Yu I.J. (2011). *In vivo* genotoxicity of silver nanoparticles after 90-day silver nanoparticle inhalation exposure. Saf. Health Work.

[73] Tiwari D.K., Jin T., Behari J. (2011). Dose-dependent *in vivo* toxicity assessment of silver nanoparticle in wistar rats. Toxicol. Mech. Methods.

[74] Rahman M.F., Wang J., Patterson T.A., Saini U.T., Robinson B.L., Newport G.D., Murdock R.C., Schlager J.J., Hussain S.M., Ali S.F. (2009). Expression of genes related to oxidative stress in the mouse brain after exposure to silver-25 nanoparticles. Toxicol. Lett..

[75] Kong B., Seog J.H., Graham L.M., Lee S.B. (2011). Experimental considerations on the cytotoxicity of nanoparticles. Nanomedicine.

[76] Diaz B., Sanchez-Espinel C., Arruebo M., Faro J., de Miguel E., Magadan S., Yague C., Fernandez-Pacheco R., Ibarra M.R., Santamaria J. (2008). Assessing methods for blood cell cytotoxic responses to inorganic nanoparticles and nanoparticle aggregates. Small.

[77] Nan A., Bai X., Son S.J., Lee S.B., Ghandehari H. (2008). Cellular uptake and cytotoxicity of silica nanotubes. Nano Lett..

[78] Chang J.S., Chang K.L., Hwang D.F., Kong Z.L. (2007). *In vitro* cytotoxicitiy of silica nanoparticles at high concentrations strongly depends on the metabolic activity type of the cell line. Environ. Sci. Technol..

[79] Kim S., Ryu D.Y. (2013). Silver nanoparticle-induced oxidative stress, genotoxicity and apoptosis in cultured cells and animal tissues. J. Appl. Toxicol..

[80] De Lima R., Seabra A.B., Duran N. (2012). Silver nanoparticles: A brief review of cytotoxicity and genotoxicity of chemically and biogenically synthesized nanoparticles. J. Appl. Toxicol..

[81] Eom H.-J., Choi J. (2010). P38 MAPK activation, DNA damage, cell cycle arrest and apoptosis as mechanisms of toxicity of silver nanoparticles in jurkat T cells. Environ. Sci. Technol..

[82] Hsin Y.H., Chen C.F., Huang S., Shih T.S., Lai P.S., Chueh P.J. (2008). The apoptotic effect of nanosilver is mediated by a ROS- and JNK-dependent mechanism involving the mitochondrial pathway in NIH3T3 cells. Toxicol. Lett..

[83] Sanpui P., Chattopadhyay A., Ghosh S.S. (2011). Induction of apoptosis in cancer cells at low silver nanoparticle concentrations using chitosan nanocarrier. ACS Appl. Mater. Interfaces.

[84] Kim S., Choi J.E., Choi J., Chung K.-H., Park K., Yi J., Ryu D.-Y. (2009). Oxidative stress-dependent toxicity of silver nanoparticles in human hepatoma cells. Toxicol. in Vitro.

[85] Liu W., Wu Y., Wang C., Li H.C., Wang T., Liao C.Y., Cui L., Zhou Q.F., Yan B., Jiang G.B. (2010). Impact of silver nanoparticles on human cells: Effect of particle size. Nanotoxicology.

[86] Braydich-Stolle L.K., Lucas B., Schrand A., Murdock R.C., Lee T., Schlager J.J., Hussain S.M., Hofmann M.-C. (2010). Silver nanoparticles disrupt GDNF/Fyn kinase signaling in spermatogonial stem cells. Toxicol. Sci..

[87] Braydich-Stolle L., Hussain S., Schlager J.J., Hofmann M.C. (2005). *In vitro* cytotoxicity of nanoparticles in mammalian germline stem cells. Toxicol. Sci..

[88] Schrand A.M., Braydich-Stolle L.K., Schlager J.J., Dai L., Hussain S.M. (2008). Can silver nanoparticles be useful as potential biological labels?. Nanotechnology.

[89] Hussain S.M., Hess K.L., Gearhart J.M., Geiss K.T., Schlager J.J. (2005). *In vitro* toxicity of nanoparticles in BRL 3A rat liver cells. Toxicol. in Vitro.

[90] Hussain S.M., Javorina A.K., Schrand A.M., Duhart H.M., Ali S.F., Schlager J.J. (2006). The interaction of manganese nanoparticles with pc-12 cells induces dopamine depletion. Toxicol. Sci..

[91] Hackenberg S., Scherzed A., Kessler M., Hummel S., Technau A., Froelich K., Ginzkey C., Koehler C., Hagen R., Kleinsasser N. (2011). Silver nanoparticles: Evaluation of DNA damage, toxicity and functional impairment in human mesenchymal stem cells. Toxicol. Lett..

[92] Liu P., Guan R., Ye X., Jiang J., Liu M., Huang G., Chen X. (2011). Toxicity of nano- and micro-sized silver particles in human hepatocyte cell line L02. J. Phys..

[93] Ahamed M., Karns M., Goodson M., Rowe J., Hussain S.M., Schlager J.J., Hong Y. (2008). DNA damage response to different surface chemistry of silver nanoparticles in mammalian cells. Toxicol. Appl. Pharmacol..

[94] Gopinath P., Gogoi S.K., Sanpui P., Paul A., Chattopadhyay A., Ghosh S.S. (2010). Signaling gene cascade in silver nanoparticle induced apoptosis. Colloids Surf. B.

[95] Miura N., Shinohara Y. (2009). Cytotoxic effect and apoptosis induction by silver nanoparticles in HeLa cells. Biochem. Biophy. Res. Commun..

[96] Li P.-W., Kuo T.-H., Chang J.-H., Yeh J.-M., Chan W.-H. (2010). Induction of cytotoxicity and apoptosis in mouse blastocysts by silver nanoparticles. Toxicol. Lett..

[97] Wei L., Tang J., Zhang Z., Chen Y., Zhou G., Xi T. (2010). Investigation of the cytotoxicity mechanism of silver nanoparticles *in vitro*. Biomed. Mater..

[98] Frohlich E., Salar-Behzadi S. (2014). Toxicological assessment of inhaled nanoparticles: Role of *in vivo*, *ex vivo*, *in vitro*, and *in silico* studies. Int. J. Mol. Sci..

[99] Zabner J., Karp P., Seiler M., Phillips S.L., Mitchell C.J., Saavedra M., Welsh M., Klingelhutz A.J. (2003). Development of cystic fibrosis and noncystic fibrosis airway cell lines. Am. J. Physiol..

[100] Worle-Knirsch J.M., Pulskamp K., Krug H.F. (2006). Oops they did it again! Carbon nanotubes hoax scientists in viability assays. Nano Lett..

[101] Casey A., Herzog E., Davoren M., Lyng F.M., Byrne H.J., Chambers G. (2007). Spectroscopic analysis confirms the interactions between single walled carbon nanotubes and various dyes commonly used to assess cytotoxicity. Carbon.

[102] Ciofani G., Danti S., D’Alessandro D., Moscato S., Menciassi A. (2010). Assessing cytotoxicity of boron nitride nanotubes: Interference with the mtt assay. Biochem. Biophys. Res. Commun..

[103] Park M.V., Annema W., Salvati A., Lesniak A., Elsaesser A., Barnes C., McKerr G., Howard C.V., Lynch I., Dawson K.A. (2009). *In vitro* developmental toxicity test detects inhibition of stem cell differentiation by silica nanoparticles. Toxicol. Appl. Pharmacol..

[104] Schulze C., Schaefer U.F., Ruge C.A., Wohlleben W., Lehr C.M. (2011). Interaction of metal oxide nanoparticles with lung surfactant protein A. Eur. J. Pharm. Biopharm..

[105] Lee Y.S., Kim D.W., Lee Y.H., Oh J.H., Yoon S., Choi M.S., Lee S.K., Kim J.W., Lee K., Song C.W. (2011). Silver nanoparticles induce apoptosis and G2/M arrest via PKCζ-dependent signaling in A549 lung cells. Arch. Toxicol..

[106] Carlson C., Hussain S.M., Schrand A.M., K. Braydich-Stolle L., Hess K.L., Jones R.L., Schlager J.J. (2008). Unique cellular interaction of silver nanoparticles: Size-dependent generation of reactive oxygen species. J. Phys. Chem. B.

[107] Nguyen K.C., Seligy V.L., Massarsky A., Moon T.W., Rippstein P., Tan J., Tayabali A.F. (2013). Comparison of toxicity of uncoated and coated silver nanoparticles. J. Phys..

[108] Oh W.-K., Kim S., Choi M., Kim C., Jeong Y.S., Cho B.-R., Hahn J.-S., Jang J. (2010). Cellular uptake, cytotoxicity, and innate immune response of silica-titania hollow nanoparticles based on size and surface functionality. ACS Nano.

[109] Kawata K., Osawa M., Okabe S. (2009). *In vitro* toxicity of silver nanoparticles at noncytotoxic doses to HepG2 human hepatoma cells. Environ. Sci. Technol..

[110] Lim D.H., Jang J., Kim S., Kang T., Lee K., Choi I.H. (2012). The effects of sub-lethal concentrations of silver nanoparticles on inflammatory and stress genes in human macrophages using cDNA microarray analysis. Biomaterials.

[111] Lewis L.N. (1993). Chemical catalysis by colloids and clusters. Chem. Rev..

[112] Gliga A.R., Skoglund S., Wallinder I.O., Fadeel B., Karlsson H.L. (2014). Size-dependent cytotoxicity of silver nanoparticles in human lung cells: The role of cellular uptake, agglomeration and Ag release. Part. Fibre Toxicol..

[113] AshaRani P.V., Hande M., Valiyaveettil S. (2009). Anti-proliferative activity of silver nanoparticles. BMC Cell Biol..

[114] Verma N.K., Conroy J., Lyons P.E., Coleman J., O’Sullivan M.P., Kornfeld H., Kelleher D., Volkov Y. (2012). Autophagy induction by silver nanowires: A new aspect in the biocompatibility assessment of nanocomposite thin films. Toxicol. Appl. Pharmacol..

[115] Gu Y.J., Cheng J., Lin C.C., Lam Y.W., Cheng S.H., Wong W.T. (2009). Nuclear penetration of surface functionalized gold nanoparticles. Toxicol. Appl. Pharmacol..

[116] Zhang M., Li J., Xing G., He R., Li W., Song Y., Guo H. (2011). Variation in the internalization of differently sized nanoparticles induces different DNA-damaging effects on a macrophage cell line. Arch. Toxicol..

[117] Haase A., Tentschert J., Jungnickel H., Graf P., Mantion A., Draude F., Plendl J., Goetz M.E., Galla S., Mašić A. (2011). Toxicity of silver nanoparticles in human macrophages: Uptake, intracellular distribution and cellular responses. J. Phys..

[118] Cronholm P., Karlsson H.L., Hedberg J., Lowe T.A., Winnberg L., Elihn K., Wallinder I.O., Moller L. (2013). Intracellular uptake and toxicity of Ag and CuO nanoparticles: A comparison between nanoparticles and their corresponding metal ions. Small.

[119] Panté N., Kann M. (2002). Nuclear pore complex is able to transport macromolecules with diameters of ~39 nm. Mol. Biol. Cell.

[120] Yuan Y., Liu C., Qian J., Wang J., Zhang Y. (2010). Size-mediated cytotoxicity and apoptosis of hydroxyapatite nanoparticles in human hepatoma HepG2 cells. Biomaterials.

[121] Jena P., Mohanty S., Mallick R., Jacob B., Sonawane A. (2012). Toxicity and antibacterial assessment of chitosan-coated silver nanoparticles on human pathogens and macrophage cells. Int. J. Nanomed..

[122] Herzog F., Clift M.J.D., Piccapietra F., Behra R., Schmid O., Petri-Fink A., Rothen-Rutishauser B. (2013). Exposure of silver-nanoparticles and silver-ions to lung cells *in vitro* at the air-liquid interface. Part. Fibre Toxicol..

[123] Chen S., Goode A.E., Sweeney S., Theodorou I.G., Thorley A.J., Ruenraroengsak P., Chang Y., Gow A., Schwander S., Skepper J. (2013). Sulfidation of silver nanowires inside human alveolar epithelial cells: A potential detoxification mechanism. Nanoscale.

[124] Schleh C., Kreyling W.G., Lehr C.M. (2013). Pulmonary surfactant is indispensable in order to simulate the *in vivo* situation. Part. Fibre Toxicol..

[125] Rivera Gil P., Oberdörster G., Elder A., Puntes V., Parak W.J. (2010). Correlating physico-chemical with toxicological properties of nanoparticles: The present and the future. ACS Nano.

[126] Rothen-Rutishauser B., Mueller L., Blank F., Brandenberger C., Muehlfeld C., Gehr P. (2008). A newly developed *in vitro* model of the human epithelial airway barrier to study the toxic potential of nanoparticles. Altex.

[127] Blank F., Rothen-Rutishauser B., Gehr P. (2007). Dendritic cells and macrophages form a transepithelial network against foreign particulate antigens. Am. J. Respir. Cell Mol. Biol..

[128] Rothen-Rutishauser B., Muhlfeld C., Blank F., Musso C., Gehr P. (2007). Translocation of particles and inflammatory responses after exposure to fine particles and nanoparticles in an epithelial airway model. Part. Fibre Toxicol..

[129] Blank F., Wehrli M., Lehmann A., Baum O., Gehr P., von Garnier C., Rothen-Rutishauser B.M. (2011). Macrophages and dendritic cells express tight junction proteins and exchange particles in an *in vitro* model of the human airway wall. Immunobiology.

[130] Xia Y., Xiong Y., Lim B., Skrabalak S.E. (2009). Shape-controlled synthesis of metal nanocrystals: Simple chemistry meets complex physics?. Angew. Chem..

[131] Warheit D.B. (2008). How meaningful are the results of nanotoxicity studies in the absence of adequate material characterization?. Toxicol. Sci..

[132] Brar S.K., Verma M. (2011). Measurement of nanoparticles by light-scattering techniques. TrAC Trends Anal. Chem..

[133] Tomaszewska E., Soliwoda K., Kadziola K., Tkacz-Szczesna B., Celichowski G., Cichomski M., Szmaja W., Grobelny J. (2013). Detection limits of dls and UV-Vis spectroscopy in characterization of polydisperse nanoparticles colloids. J. Nanomater..

[134] Khlebtsov B.N., Khlebtsov N.G. (2011). On the measurement of gold nanoparticle sizes by the dynamic light scattering method. Colloid J..

[135] Milne J.L.S., Borgnia M.J., Bartesaghi A., Tran E.E.H., Earl L.A., Schauder D.M., Lengyel J., Pierson J., Patwardhan A., Subramaniam S. (2013). Cryo-electron microscopy—A primer for the non-microscopist. FEBS J..

[136] Elechiguerra J.L., Larios-Lopez L., Liu C., Garcia-Gutierrez D., Camacho-Bragado A., Yacaman M.J. (2005). Corrosion at the nanoscale: The case of silver nanowires and nanoparticles. Chem. Mater..

[137] Laban G., Nies L.F., Turco R.F., Bickham J.W., Sepulveda M.S. (2010). The effects of silver nanoparticles on fathead minnow (*Pimephales promelas*) embryos. Ecotoxicology.

[138] Beer C., Foldbjerg R., Hayashi Y., Sutherland D.S., Autrup H. (2012). Toxicity of silver nanoparticles—Nanoparticle or silver ion?. Toxicol. Lett..

[139] Lee Y.J., Kim J., Oh J., Bae S., Lee S., Hong I.S., Kim S.H. (2012). Ion-release kinetics and ecotoxicity effects of silver nanoparticles. Environ. Toxicol. Chem..

[140] Chappell M.A., Miller L.F., George A.J., Pettway B.A., Price C.L., Porter B.E., Bednar A.J., Seiter J.M., Kennedy A.J., Steevens J.A. (2011). Simultaneous dispersion-dissolution behavior of concentrated silver nanoparticle suspensions in the presence of model organic solutes. Chemosphere.

[141] Liu J., Wang Z., Liu F.D., Kane A.B., Hurt R.H. (2012). Chemical transformations of nanosilver in biological environments. ACS Nano.

[142] Glover R.D., Miller J.M., Hutchison J.E. (2011). Generation of metal nanoparticles from silver and copper objects: Nanoparticle dynamics on surfaces and potential sources of nanoparticles in the environment. ACS Nano.

[143] Chen S., Theodorou I.G., Goode A.E., Gow A., Schwander S., Zhang J., Chung K.F., Tetley T.D., Shaffer M.S., Ryan M.P. (2013). High-resolution analytical electron microscopy reveals cell culture media-induced changes to the chemistry of silver nanowires. Environ. Sci. Technol..

[144] Levard C., Hotze E.M., Lowry G.V., Brown G.E. (2012). Environmental transformations of silver nanoparticles: Impact on stability and toxicity. Environ. Sci. Technol..

[145] Levard C., Reinsch B.C., Michel F.M., Oumahi C., Lowry G.V., Brown G.E. (2011). Sulfidation processes of PVP-coated silver nanoparticles in aqueous solution: Impact on dissolution rate. Environ. Sci. Technol..

[146] Reinsch B.C., Levard C., Li Z., Ma R., Wise A., Gregory K.B., Brown G.E., Lowry G.V. (2012). Sulfidation of silver nanoparticles decreases escherichia coli growth inhibition. Environ. Sci. Technol..

[147] Bondarenko O., Ivask A., Kakinen A., Kurvet I., Kahru A. (2013). Particle-cell contact enhances antibacterial activity of silver nanoparticles. PLoS One.

[148] Monopoli M.P., Aberg C., Salvati A., Dawson K.A. (2012). Biomolecular coronas provide the biological identity of nanosized materials. Nat. Nano.

[149] Lynch I., Dawson K.A. (2008). Protein-nanoparticle interactions. Nano Today.

[150] Cedervall T., Lynch I., Lindman S., Berggård T., Thulin E., Nilsson H., Dawson K.A., Linse S. (2007). Understanding the nanoparticle–protein corona using methods to quantify exchange rates and affinities of proteins for nanoparticles. Proc. Natl. Acad. Sci. USA.

[151] Powers C.M., Badireddy A.R., Ryde I.T., Seidler F.J., Slotkin T.A. (2011). Silver nanoparticles compromise neurodevelopment in PC12 cells: Critical contributions of silver ion, particle size, coating, and composition. Environ. Health Perspect..

[152] Kittler S., Greulich C., Gebauer J.S., Diendorf J., Treuel L., Ruiz L., Gonzalez-Calbet J.M., Vallet-Regi M., Zellner R., Koller M. (2010). The influence of proteins on the dispersability and cell-biological activity of silver nanoparticles. J. Mater. Chem..

[153] Yen H.J., Hsu S.H., Tsai C.L. (2009). Cytotoxicity and immunological response of gold and silver nanoparticles of different sizes. Small.

[154] Gnanadhas D.P., Ben Thomas M., Thomas R., Raichur A.M., Chakravortty D. (2013). Interaction of silver nanoparticles with serum proteins affects their antimicrobial activity *in vivo*. Antimicrob. Agents Chemother..

[155] Bell R.A., Kramer J.R. (1999). Structural chemistry and geochemistry of silver-sulfur compounds: Critical review. Environ. Toxicol. Chem..

[156] Creuwels L.A., van Golde L.M., Haagsman H.P. (1997). The pulmonary surfactant system: Biochemical and clinical aspects. Lung.

[157] Johansson J., Curstedt T., Robertson B. (1994). The proteins of the surfactant system. Eur. Respir. J..

[158] Goerke J. (1998). Pulmonary surfactant: Functions and molecular composition. Biochim. Biophys. Acta.

[159] Porter D., Sriram K., Wolfarth M., Jefferson A., Schwegler-Berry D., Andrew M.E., Castranova V. (2008). A biocompatible medium for nanoparticle dispersion. Nanotoxicology.

[160] Sager T.M., Porter D.W., Robinson V.A., Lindsley W.G., Schwegler-Berry D.E., Castranova V. (2007). Improved method to disperse nanoparticles for *in vitro* and *in vivo* investigation of toxicity. Nanotoxicology.

[161] MacCuspie R.I., Allen A.J., Hackley V.A. (2011). Dispersion stabilization of silver nanoparticles in synthetic lung fluid studied under *in situ* conditions. Nanotoxicology.

[162] Bakshi M.S., Zhao L., Smith R., Possmayer F., Petersen N.O. (2008). Metal nanoparticle pollutants interfere with pulmonary surfactant function *in vitro*. Biophys. J..

[163] Stringer B., Kobzik L. (1996). Alveolar macrophage uptake of the environmental particulate titanium dioxide: Role of surfactant components. Am. J. Respir. Cell Mol. Biol..

[164] Konduru N.V., Tyurina Y.Y., Feng W., Basova L.V., Belikova N.A., Bayir H., Clark K., Rubin M., Stolz D., Vallhov H. (2009). Phosphatidylserine targets single-walled carbon nanotubes to professional phagocytes *in vitro* and *in vivo*. PLoS One.

[165] Gasser M., Wick P., Clift M.J., Blank F., Diener L., Yan B., Gehr P., Krug H.F., Rothen-Rutishauser B. (2012). Pulmonary surfactant coating of multi-walled carbon nanotubes (MWCNTs) influences their oxidative and pro-inflammatory potential *in vitro*. Part. Fibre Toxicol..

[166] Herzog E., Byrne H.J., Davoren M., Casey A., Duschl A., Oostingh G.J. (2009). Dispersion medium modulates oxidative stress response of human lung epithelial cells upon exposure to carbon nanomaterial samples. Toxicol. Appl. Pharmacol..

[167] Zhao F., Zhao Y., Liu Y., Chang X., Chen C., Zhao Y. (2011). Cellular uptake, intracellular trafficking, and cytotoxicity of nanomaterials. Small.

[168] Nel A.E., Madler L., Velegol D., Xia T., Hoek E.M., Somasundaran P., Klaessig F., Castranova V., Thompson M. (2009). Understanding biophysicochemical interactions at the nano-bio interface. Nat. Mater..

[169] Verma A., Stellacci F. (2010). Effect of surface properties on nanoparticle–cell interactions. Small.

[170] Mailander V., Landfester K. (2009). Interaction of nanoparticles with cells. Biomacromolecules.

[171] Conner S.D., Schmid S.L. (2003). Regulated portals of entry into the cell. Nature.

[172] Soldati T., Schliwa M. (2006). Powering membrane traffic in endocytosis and recycling. Nat. Rev..

[173] Harush-Frenkel O., Debotton N., Benita S., Altschuler Y. (2007). Targeting of nanoparticles to the clathrin-mediated endocytic pathway. Biochem. Biophys. Res. Commun..

[174] Greulich C., Diendorf J., Simon T., Eggeler G., Epple M., Köller M. (2011). Uptake and intracellular distribution of silver nanoparticles in human mesenchymal stem cells. Acta Biomater..

[175] Geiser M., Rothen-Rutishauser B., Kapp N., Schurch S., Kreyling W., Schulz H., Semmler M., Im Hof V., Heyder J., Gehr P. (2005). Ultrafine particles cross cellular membranes by nonphagocytic mechanisms in lungs and in cultured cells. Environ. Health Perspect..

[176] Rimai D.S., Quesnel D.J., Busnaina A.A. (2000). The adhesion of dry particles in the nanometer to micrometer-size range. Colloids Surf. A.

[177] Lehmann A.D., Parak W.J., Zhang F., Ali Z., Röcker C., Nienhaus G.U., Gehr P., Rothen-Rutishauser B. (2010). Fluorescent–magnetic hybrid nanoparticles induce a dose-dependent increase in proinflammatory response in lung cells *in vitro* correlated with intracellular localization. Small.

[178] Geiser M., Casaulta M., Kupferschmid B., Schulz H., Semmler-Behnke M., Kreyling W. (2008). The role of macrophages in the clearance of inhaled ultrafine titanium dioxide particles. Am. J. Respir. Cell Mol. Biol..

[179] Johnston H.J., Semmler-Behnke M., Brown D.M., Kreyling W., Tran L., Stone V. (2010). Evaluating the uptake and intracellular fate of polystyrene nanoparticles by primary and hepatocyte cell lines *in vitro*. Toxicol. Appl. Pharmacol..

[180] Motskin M., Wright D.M., Muller K., Kyle N., Gard T.G., Porter A.E., Skepper J.N. (2009). Hydroxyapatite nano and microparticles: Correlation of particle properties with cytotoxicity and biostability. Biomaterials.

[181] Wang L., Liu Y., Li W., Jiang X., Ji Y., Wu X., Xu L., Qiu Y., Zhao K., Wei T. (2011). Selective targeting of gold nanorods at the mitochondria of cancer cells: Implications for cancer therapy. Nano Lett..

[182] Panyam J., Zhou W.Z., Prabha S., Sahoo S.K., Labhasetwar V. (2002). Rapid endo-lysosomal escape of poly(dl-lactide-co-glycolide) nanoparticles: Implications for drug and gene delivery. FASEB J..

[183] Ji Z., Wang X., Zhang H., Lin S., Meng H., Sun B., George S., Xia T., Nel A.E., Zink J.I. (2012). Designed synthesis of CeO_2_ nanorods and nanowires for studying toxicological effects of high aspect ratio nanomaterials. ACS Nano.

[184] Chu Z., Zhang S., Zhang B., Zhang C., Fang C.-Y., Rehor I., Cigler P., Chang H.-C., Lin G., Liu R. Unambiguous observation of shape effects on cellular fate of nanoparticles. Sci. Rep..

[185] Panariti A., Miserocchi G., Rivolta I. (2012). The effect of nanoparticle uptake on cellular behavior: Disrupting or enabling functions?. Nanotechnol. Sci. Appl..

[186] Dausend J., Musyanovych A., Dass M., Walther P., Schrezenmeier H., Landfester K., Mailänder V. (2008). Uptake mechanism of oppositely charged fluorescent nanoparticles in HeLa cells. Macromol. Biosci..

[187] Brandenberger C., Mühlfeld C., Ali Z., Lenz A.-G., Schmid O., Parak W.J., Gehr P., Rothen-Rutishauser B. (2010). Quantitative evaluation of cellular uptake and trafficking of plain and polyethylene glycol-coated gold nanoparticles. Small.

[188] Donaldson K., Murphy F., Schinwald A., Duffin R., Poland C.A. (2011). Identifying the pulmonary hazard of high aspect ratio nanoparticles to enable their safety-by-design. Nanomedicine.

[189] Schinwald A., Murphy F.A., Prina-Mello A., Poland C.A., Byrne F., Movia D., Glass J.R., Dickerson J.C., Schultz D.A., Jeffree C.E. (2012). The threshold length for fiber-induced acute pleural inflammation: Shedding light on the early events in asbestos-induced mesothelioma. Toxicol. Sci..

[190] Teeguarden J.G., Hinderliter P.M., Orr G., Thrall B.D., Pounds J.G. (2007). Particokinetics *in vitro*: Dosimetry considerations for *in vitro* nanoparticle toxicity assessments. Toxicol. Sci..

[191] Daskalakis K.D., O’Connor T.P., Crecelius E.A. (1997). Evaluation of digestion procedures for determining silver in mussels and oysters. Environ. Sci. Technol..

[192] Singh R.P., Ramarao P. (2012). Cellular uptake, intracellular trafficking and cytotoxicity of silver nanoparticles. Toxicol. Lett..

[193] Pratsinis A., Hervella P., Leroux J.-C., Pratsinis S.E., Sotiriou G.A. (2013). Toxicity of silver nanoparticles in macrophages. Small.

[194] Park E.J., Yi J., Kim Y., Choi K., Park K. (2010). Silver nanoparticles induce cytotoxicity by a trojan-horse type mechanism. Toxicol. in Vitro.

[195] Vanwinkle B.A., de Mesy Bentley K.L., Malecki J.M., Gunter K.K., Evans I.M., Elder A., Finkelstein J.N., Oberdorster G., Gunter T.E. (2009). Nanoparticle (NP) uptake by type I alveolar epithelial cells and their oxidant stress response. Nanotoxicology.

[196] Muller K.H., Kulkarni J., Motskin M., Goode A., Winship P., Skepper J.N., Ryan M.P., Porter A.E. (2010). pH-Dependent toxicity of high aspect ratio ZnO nanowires in macrophages due to intracellular dissolution. ACS Nano.

